# Tau Aggregate Imaging and Transcriptomics of Alzheimer's Disease Brain at Different Stages of Disease

**DOI:** 10.1002/advs.77453

**Published:** 2026-08-31

**Authors:** Elizabeth A. English, Dorothea Böken, Matthew W. Cotton, Matthew R. Cheetham, Johanna S. Jackson, Georg Meisl, John S. H. Danial, Paul M. Matthews, Nurun N. Fancy, David Klenerman

**Affiliations:** ^1^ Yusuf Hamied Department of Chemistry University of Cambridge Cambridge UK; ^2^ UK Dementia Research Institute at University of Cambridge Cambridge UK; ^3^ Department of Brain Sciences Imperial College London London UK; ^4^ UK Dementia Research Institute Imperial College London London UK; ^5^ School of Physics and Astronomy University of St Andrews St Andrews UK; ^6^ Rosalind Franklin Institute Didcot UK

## Abstract

Tau aggregation plays a critical role in the development and progression of Alzheimer's disease (AD). Tau aggregates of different sizes and shapes are formed, which ultimately lead to the deposition of fibrillar tangles. We used single‐molecule techniques to characterize tau aggregates in the middle temporal gyrus and somatosensory cortex in post‐mortem brain homogenates at different Braak stages from patients with AD. Total and phosphorylated tau aggregates increased dramatically in late Braak stages. The aggregates showed greater multi‐site phosphorylation with increased Braak stage, but there was only a moderate change in the aggregate size distribution. Paired single nuclei transcriptomic analyses provided evidence for greater pro‐inflammatory microglial and complement pathway activation with increasing phosphorylated tau aggregate concentration and length. Based on this correlation, we hypothesize a cascade of disease progression in which microglial inflammation induces tau aggregation in neighboring neurons that, in turn, further enhances inflammation and the spread of tau pathology to increase the concentration of small tau aggregates.

## Graphical abstract

The protein aggregates and gene expression in the middle temporal gyrus (MTG) and somatosensory cortex (SOM) of the postmortem brains of 13 Alzheimer's disease patients were studied in detail, revealing that small hyperphosphorylated tau aggregates increase with Braak stage driven by microglial inflammation.

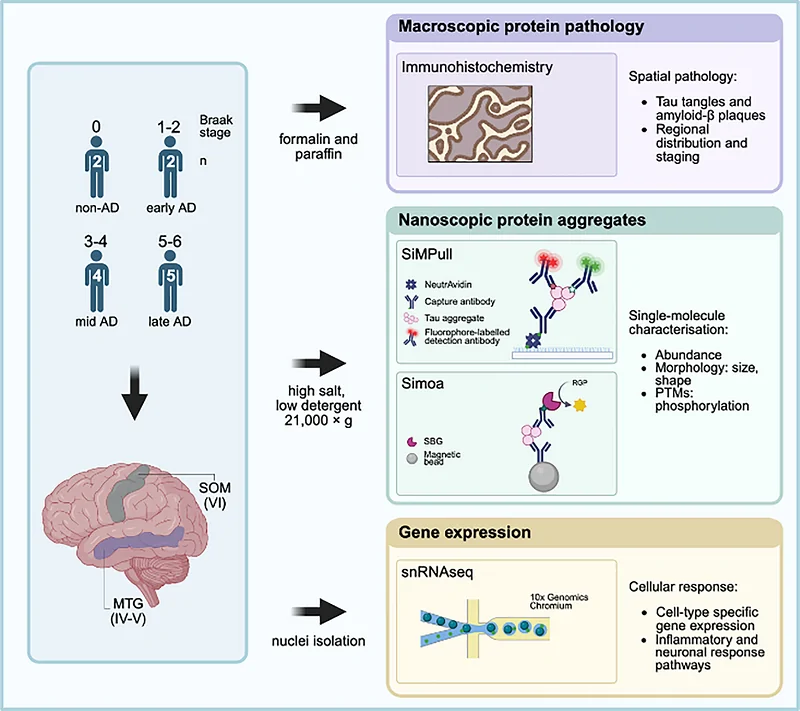

## 1 Introduction

Alzheimer's disease (AD) is one of the greatest healthcare challenges facing our society. It is characterized by the formation of extracellular plaques of amyloid‐beta and intracellular neurofibrillary tangles (NFTs) of the microtubule‐binding protein tau. The formation of tau tangles correlates more strongly with synaptic loss and cognitive decline than amyloid plaques [1]. Therefore, there is great interest in characterizing the tau aggregates across disease progression and in understanding the mechanisms by which they form and evolve. While the structure of the end‐stage fibrils has recently been determined using cryo‐EM [2, 3], it has been difficult to study the early‐stage small tau aggregates that form in human tissue due to their greater heterogeneity in size and shape, low abundance and lack of suitable characterization methods. However, these early aggregates are particularly important since there is evidence that they are cytotoxic [4]. To address this, we have developed sensitive single aggregate methods to characterize the number, size and shape of tau aggregates and their post‐translational modifications [5]. In this work, we apply these methods to characterize the tau aggregates that form in post‐mortem AD brain with increasing progression of disease. This new information provides an opportunity to rule out certain postulated mechanisms of tau aggregation and determine which mechanisms are likely to occur in the human disease, noting that it will be impossible to directly prove which mechanisms occur in vivo in humans.

Hyperphosphorylation is widely recognized as an important step in the aggregation of tau. It reduces tau's affinity for microtubules, leading to its dissociation and increasing its propensity to aggregate. In AD, tau is hyperphosphorylated with an average of 5–9 phosphate groups per tau protein, compared to 2–3 phosphates on healthy tau [6]. Hyperphosphorylation of tau can also spontaneously induce aggregation in vitro without the need of any further additives such as heparin [7]. Replication‐competent tau aggregates are known as tau seeds. One striking observation about tau aggregation in humans is that the number of seeds and deposited tau measured using immunohistochemistry increases exponentially with time [8]. Mathematical modelling suggests that this exponential increase is in both the number of cells with tau aggregates and the number of tau aggregates overall, with a doubling time of 5 years [8]. This exponential increase in the deposited tau is in marked contrast to the more linear increase in deposited amyloid‐beta observed in AD [9]. Furthermore, our recent quantitative measurements of tau and amyloid‐beta aggregates in prefrontal cortex brain homogenate show that there are about 100‐fold more tau aggregates than amyloid‐beta aggregates in control brain, increasing to 6000‐fold more in Braak stage 6 [10]. Tau aggregates are therefore the dominant aggregate in AD brains, and understanding what triggers tau aggregation and the mechanism by which tau aggregates increase in the brain as disease progresses are key unaddressed questions in the field.

Accumulating evidence suggests that these processes extend beyond tau itself. Neuroinflammatory pathways have been implicated in the propagation of tau pathology, with glial activation and enhanced complement cascade activity proposed to exacerbate tau pathology [11] and contribute to synaptic loss [12]. In parallel, dysregulation of neuronal tau‐associated kinases and phosphatases is thought to promote pathological tau hyperphosphorylation [13]. However, how these pathways interact to drive tau aggregation in the human brain remains unclear. To address this, we employ a multi‐omics approach combining bulk and single‐nucleus transcriptomic data from the same set of postmortem brains, enabling systematic identification of genes and pathways associated with changes in tau aggregation.

**FIGURE 1:**
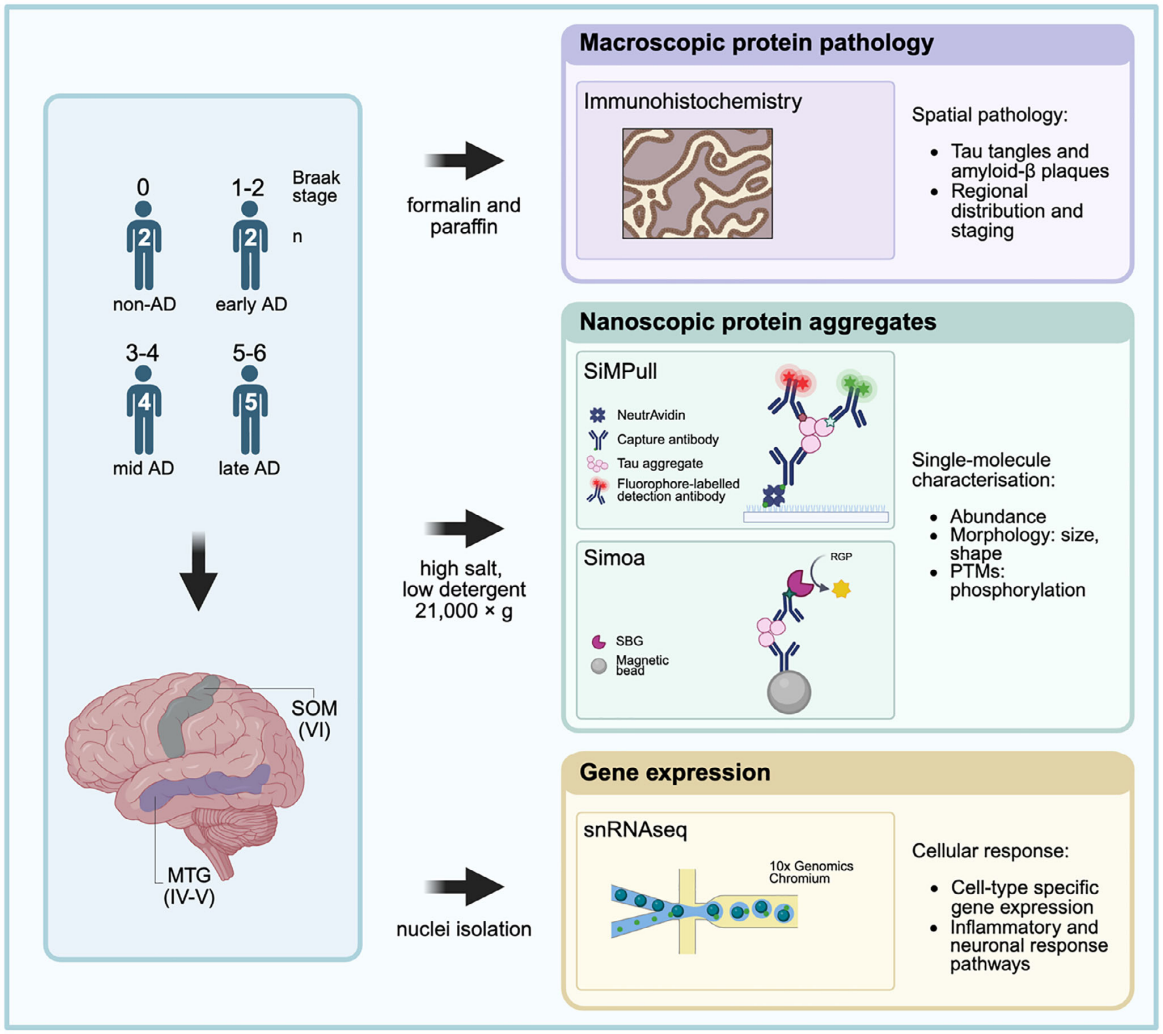
Sample characteristics and experimentation methods. This study examined brain samples from 13 donors spanning Alzheimer's disease (AD) Braak stages, 0–6. Each of the donors provided samples of their middle temporal gyrus (MTG) somatosensory cortex (SOM). Formalin and paraffin‐fixed sections of these regions were used for immunohistochemistry analysis of the area covered by protein aggregates. Fresh frozen brain tissue from the same donor brains was homogenized to extract brain proteins into liquid for several experiments. Single molecule array (Simoa) assays measured aggregate concentration. This method is based on the capture of individual beads in microwells and detecting the fluorescence product produced by Streptavidin β‐galactosidase (SBG) in each well. Single molecule pull‐down (SiMPull) microscopy measured aggregate size, shape and number, including aggregates that had post‐translational modifications (PTMs) and were phosphorylated. Bulk RNA sequencing was performed on an RNAase‐free tissue homogenate and single nucleus RNA sequencing (snRNAseq) experiments measured gene expression levels in individual nuclei of glia and neurons also extracted from frozen sections of the same brains. Bicinchoninic acid (BCA) assays measured total protein concentration of the homogenates to normalize other experiments against.

Here, we integrate complementary single‐molecule, immunohistochemistry and transcriptomic approaches to characterize tau aggregates spanning nanoscopic assemblies to insoluble fibrils across Alzheimer's disease Braak stages (Figure 1). We focus on two regions with well‐defined differences in the onset of tau pathology during disease progression. According to traditional immunohistochemistry observations, the middle temporal gyrus (MTG) is affected by NFTs from Braak stage 3 onwards, whereas the somatosensory cortex (SOM) remains largely unaffected until the most advanced stages (Braak 5–6). The use of these regions enables direct comparison of tau aggregate properties at different stages of disease progression within the same brain. By combining this single‐molecule data with transcriptomics analysis, this study correlates tau aggregate properties with molecular pathways at different stages of AD to gain new insights into possible mechanisms of disease.

## 2 Results

### 2.1 Postmortem Cohort Characteristics Across Alzheimer's Disease Braak Stages

Postmortem flash‐frozen brain samples (*n* = 13 donors, MTG and SOM, spanning AD Braak stages 0–6, Table 1 and Table S1), were obtained from the London Neurodegenerative Diseases Brain Bank. Braak stage 0–2 donors were younger than and Braak stage 5–6 donors (*, *p* = 0.03 Wilcoxon rank‐sum test, Table S1), so age cannot be excluded as a contributor to changes differences observed between early and late Braak stage samples in this study. ApoE status, sex and post‐mortem delay between the brains were not significantly different between Braak groupings (*p* > 0.08 Wilcoxon rank‐sum test, Table S1). All donors showed absence of Lewy bodies in histopathology (Braak LB score = 0) and no evidence of TREM2 mutations.

**TABLE 1:** Clinical characteristics of brain donors in this study. More information per donor, including cognitive status with a summary per Braak stage groupings of 0, 1–2, 3–4 and 5–6 in Table S1.

Braak stages	0	1	2	3	4	5	6
Donor sample size	2	1	1	3	1	3	2
Sex (F, M (%))	0, 100	0, 100	100, 0	66.6, 33.3	0, 100	66.6, 33.3	50, 50
Age at death, years (mean ±)	75 ± 2	81	76	81 ± 13	87	84.3 ± 1.3	84.5 ± 1.5
Postmortem delay, hours (mean ±)	17 ± 6	10	22	31 ± 22	19	17.5 ± 5.5	15 ± 5
ApoE genotype (ApoE 3/3, 3/4 (%))	100, 0	100, 0	100, 0	66.6, 33.3	0, 100	33.3, 66.6	0, 100

### 2.2 Immunohistochemistry Reveals Amyloid Deposition Preceding a Significant Increase in Tau Pathology

**FIGURE 2:**
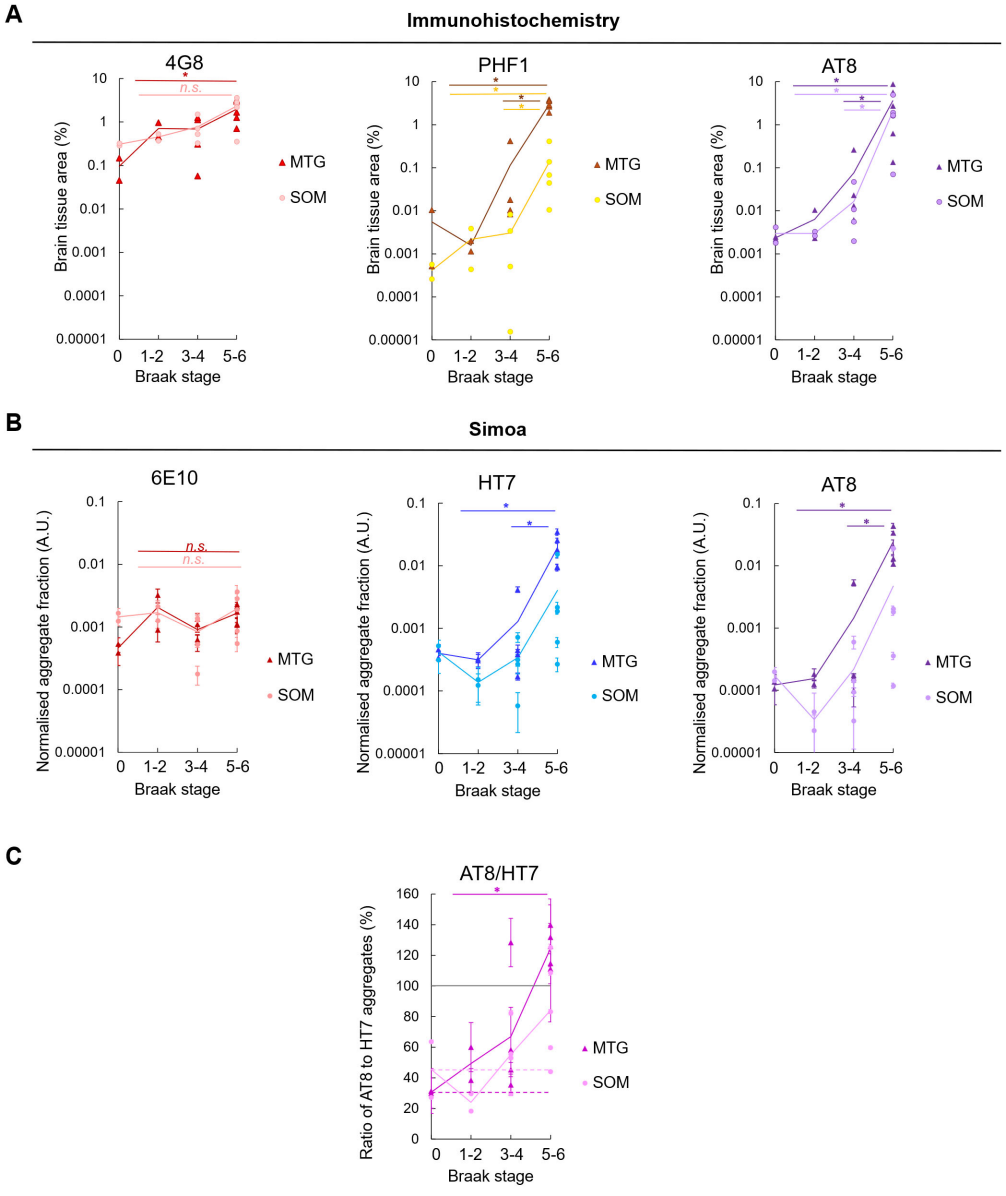
Immunohistochemistry staining of fixed brain tissue and aggregate‐specific Simoa of brain homogenate. (A) Proportion of brain tissue area stained for amyloid‐beta or phosphorylated tau between postmortem tissue donors with Braak stage grouping, as observed by immunohistochemistry. 4G8 antibodies were used for amyloid‐beta staining. For phosphorylated tau, AT8 and PHF1 were used. *n* = 13 donors. (B) Normalized fraction of brain homogenate occupied by amyloid‐beta (6E10), tau (HT7) or phosphorylated tau (AT8) aggregates, calculated by dividing Simoa‐derived aggregated monomer concentrations by total protein concentration of brain homogenate from BCA assay. (C) Ratio of AT8 to HT7 in B as an approximate measure of the extent of phosphorylation of tau aggregates. "MTG" denotes middle temporal gyrus, and "SOM," somatosensory cortex. Wilcoxon rank‐sum test between Braak stage groups 0–2, 3–4 and 5–6, ^*^ *p* < 0.05; n.s., nonsignificant. *n* = 13 donors. Each spot represents mean average results from one donor. Error bars denote ± 1 absolute error from standard errors, incorporating 3 Simoa inter‐plate repeats and 2 BCA intra‐plate repeats.

Immunohistochemistry was carried out on MTG and SOM regions to measure the areas of amyloid‐beta (4G8) and phosphorylated tau (AT8, PHF1) aggregates (Table S2). We observed slightly higher amyloid‐beta area in Braak stage 1–2 compared to Braak stage 0 in both brain regions and then a steady slight increase to Braak 6 (Figure 2A). There was a large increase in p‐tau area in samples from late Braak stages. In the MTG, amyloid‐beta aggregate area differed significantly between early (Braak 0–2) and late (Braak 5–6) stages; no significant differences were observed between intermediate stages or in the SOM. By comparison, phosphorylated tau areas were significantly increased between Braak 0–2 and 5–6, as well as between Braak 3–4 and 5–6, in both regions. Overall, these data show an expected early AD‐associated increase in amyloid‐beta deposits with a much greater increase in tau aggregates at greater Braak stages and in the MTG than the SOM, as expected by Braak staging literature [14]. Consistent trends with lower maximum aggregate areas and more modest Braak stage differences in the white matter (Figure S1).

### 2.3 Nanoscopic Tau Aggregates are Present in Healthy Control Brain and Increase Significantly at Later Braak Stages

**FIGURE 3:**
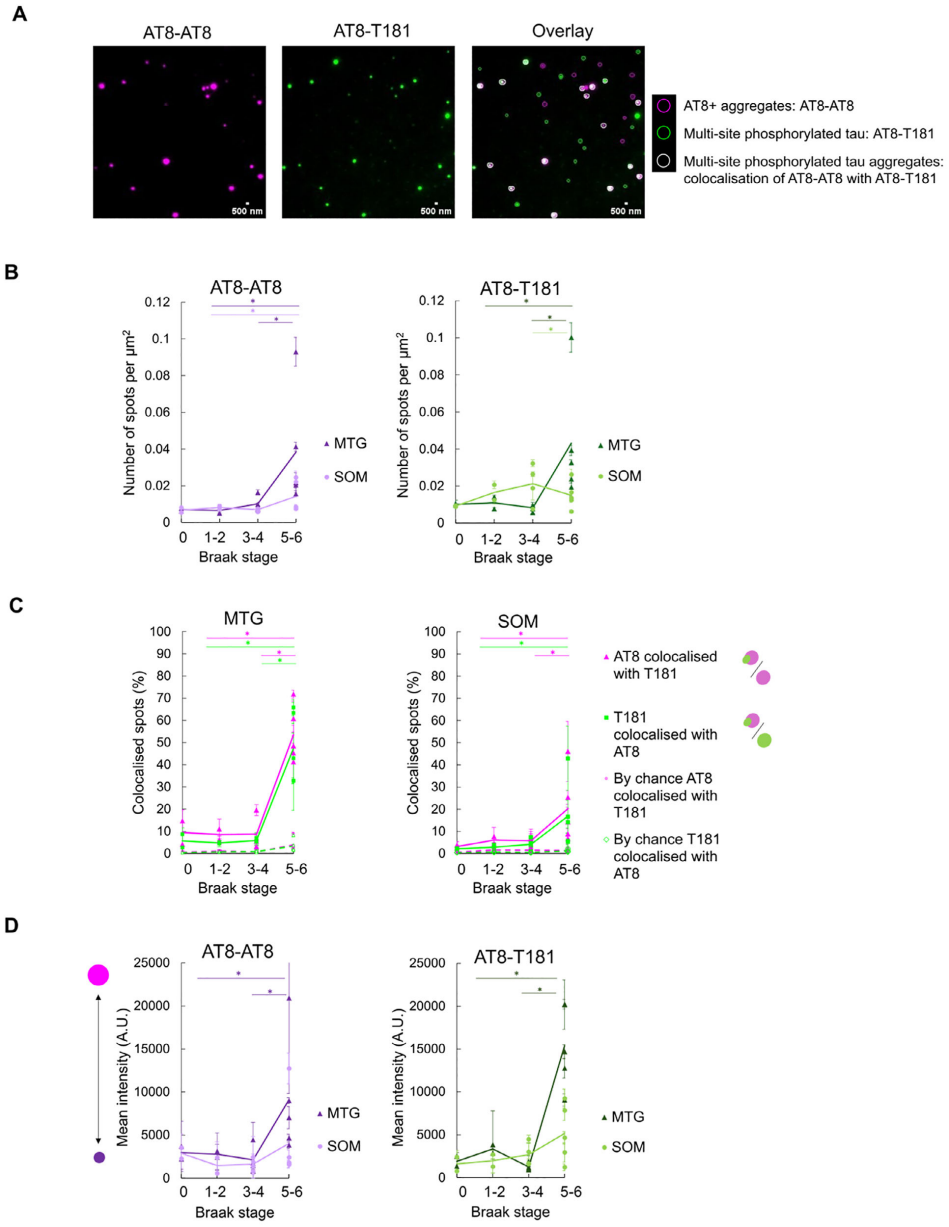
Colocalization imaging showed increased multi‐site phosphorylated tau aggregation with AD, in aggregate quantity and size. (A) Representative images of middle temporal gyrus (MTG) homogenate, observing AT8‐AT8 under 638 nm laser (shown in magenta), AT8‐T181 under 488 nm laser (shown in green), and colocalized spots (shown in white) by overlaying the two channels. AT8‐AT8 refers to AT8+ p‐tau aggregates. AT8‐T181 refers to multi‐site phosphorylated tau: phosphorylated at AT8 and T181 sites and is not necessarily aggregated. Colocalized refers to multi‐site phosphorylated tau aggregates: tau aggregates that are phosphorylated at AT8 and T181 sites. (B) Quantity of phosphorylated tau aggregates from donor middle temporal gyrus (MTG) and somatosensory cortex (SOM) as measured by the diffraction‐limited number of fluorescent spots per micron squared, normalized to total protein per homogenate sample as measured by BCA assay. (C) Proportion of colocalized spots per image as a proxy for proportion of tau aggregates phosphorylated at both AT8 and T181 sites, calculated by dividing quantity of AT8‐AT8 and AT8‐T181 colocalized spots by total AT8‐AT8 or AT8‐T181 spots respectively. Colocalized by chance percentages were calculated by rotating image color channels 180° from each other and recounting colocalized number, then dividing by colocalized number from nonrotated images. D: Mean fluorescent spot intensity, in arbitrary units, as a proxy for phosphorylated tau complex size. B‐D: *n* = 13 donors. Each spot represents mean average results from one donor, and error bars denote ± 1 absolute error from standard errors, incorporating intra‐plate error between 9 FOVs per experiment, and inter‐plate error between 3 experimental repeats. B also incorporates standard error from 2 BCA intra‐plate repeats. Wilcoxon rank‐sum test between Braak stage groups 0–2, 3–4 and 5–6, ^*^ *p* < 0.05.

We characterized the nanoscopic tau aggregates in these brains using single aggregate methods applied to homogenized fresh‐frozen brain tissue. This enabled detection of small aggregates that would be undetectable with low‐magnification immunohistochemistry. For both single‐aggregate approaches in this study, the single‐molecule array (Simoa) and the single‐molecule pull‐down (SiMPull) (Figure 3), capture and detection antibodies with the same epitope targets were applied in a “sandwich” to detect aggregates and not monomer. In previous work, we have tested monomer‐mimicking peptides with SiMPull [5] and performed denaturation experiments using the Simoa [10] to confirm that these methods detect tau aggregates not monomers. With Simoa, we compared the amount of amyloid‐beta, tau and phosphorylated tau aggregates across Braak stages and the two brain regions, normalized to the total protein in the sample (Figure 2B).

The amount of small amyloid‐beta aggregates did not change significantly with Braak stage (*p* > 0.1 Wilcoxon rank‐sum), unlike tau and p‐tau: significantly more tau and p‐tau aggregates were found in homogenate from MTG of late (5‐6) Braak stage donors compared with earlier Braak stages but not from the SOM (Figure 2B). The ratio of AT8 to HT7 for tau aggregates, increased with Braak stage in both regions from approximately 20% in non‐AD controls, to around 120% (Figure 2C).

### 2.4 Nanoscopic Tau Aggregates Become Increasingly Multi‐phosphorylated With Disease Progression

We then visualized tau proteins captured by single‐molecule pulldown, SiMPull, with diffraction‐limited fluorescence microscopy. In SiMPull, the capture antibody was anchored to a glass coverslip, and the detection antibody was fluorophore‐conjugated, for visualization with total internal reflection fluorescence (TIRF) microscopy [15]. Colocalization microscopy assessed the composition of the tau aggregates by detecting the presence of multiple phosphorylated tau monomers in the same tau aggregate. As well as studying phosphorylation at S202 and T205 using AT8 capture and AT8 detection, a second detection antibody with a different fluorophore‐conjugated dye was added, T181. Colocalizations between these fluorescent spots indicated tau aggregates with at least two AT8 phosphorylated sites and at least one phosphorylated T181 site, enabling us to determine an approximate fraction of tau aggregates that were multi‐phosphorylated (Figure 3A,B). Confirming Simoa findings, phosphorylated tau aggregate quantities were found to significantly increase towards late Braak stages (Figure 3B). There were significant increases in the fraction of colocalized spots, denoting tau aggregates phosphorylated at both AT8 and T181 sites, for the MTG and SOM (Figure 3C). In the MTG, the fraction of colocalized spots increased from less than 10% before Braak stage 3 to 30%–70% after Braak stage 3. In contrast, the increase in the SOM occurred at Braak stage 5, and only reached 10%–40%.

In the SiMPull measurements, the fluorescence intensity of a spot reflects the number of fluorophore‐labelled detection antibodies bound. Spot brightness therefore provides a relative measure of aggregate phosphorylation. Because larger aggregates may also contain more tau molecules and consequently bind more detection antibodies, fluorescence intensity must be interpreted alongside the aggregate‐size measurements.

**FIGURE 4:**
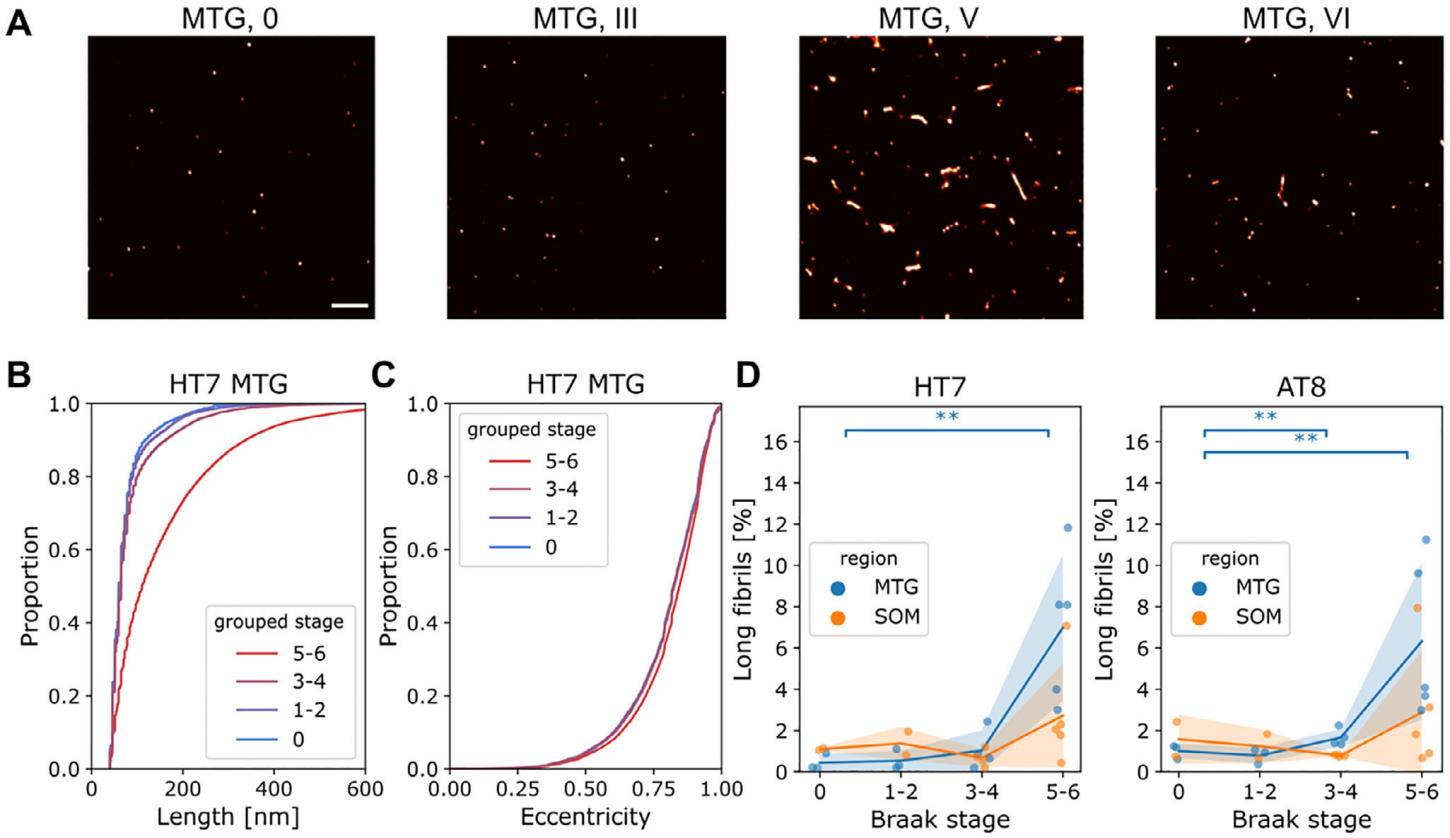
Super‐resolution imaging identified a diverse population of tau aggregate sizes and shapes across Braak stages, with a greater proportion of long fibrils at Braak 5–6. (A) Representative DNA‐PAINT images of AT8‐positive tau aggregates in MTG at different Braak stages. Scale bar = 1 µm. (B) Cumulative frequency plot of aggregate length (HT7 in MTG). (C) Cumulative frequency plot of aggregate eccentricity, with 0 representing completely circular clusters and 1 representing completely linear clusters. (D) Proportion of long, fibrillar‐shaped aggregates (>250 nm length, >0.9 eccentricity). Each spot represents the average result from one patient, shading indicates the S.D. between patients. Wilcoxon rank‐sum test with Holm correction between Braak stage groups 0–2, 3–4 and 5–6. ^**^ *p* < 0.01. Only significant comparisons are shown. (B–D) *n* = 13 donors.

We therefore performed a more detailed analysis of the mean, maximum and minimum brightness of non‐colocalized and colocalized spots (Figure S2). This showed that the average brightness of the colocalized aggregates was significantly higher than the non‐colocalized aggregates at all Braak stages (Figure S2A,B). The increase in average aggregate brightness with Braak stage therefore occurred due to an increase in the fraction of multi‐phosphorylated aggregates. Consistent with this, the proportion of bright (>500 arbitrary units of intensity) multi‐phosphorylated aggregates was significantly greater at Braak stage 5–6 compared to earlier Braak stages in the MTG homogenate, but not in the SOM (Figure S2C). The maximum intensity increased with late Braak stage, suggesting that the most highly multi‐phosphorylated aggregates are formed at late disease (Figure S2D). The minimum intensity of the non‐colocalized spots stayed at about 100 units at all Braak stage, the level of a single antibody, and was 10–100 fold less than the colocalized spots, in agreement with colocalized aggregates being brighter (Figure S2). As shown in the next section, the proportional increase in average aggregate size between Braak stage 0 and Braak stage 5–6 (approximately 2‐fold, Figure 4B) is much smaller than the proportional increase in the number of phosphorylated tau molecules per aggregate (up to 100‐fold, Figure 3D). This suggests that there is increased phosphorylation of aggregates at late Braak stages and the change observed is not simply due to the formation of larger aggregates.

Relative to the fluorescence intensity of a single detection antibody, AT8‐positive aggregates in the MTG were approximately 30‐fold brighter at early Braak stages and approximately 100‐fold brighter by Braak stage 6, while there was no significant change in the SOM with disease stage (Figure 3D). For T181, the aggregates were about 20‐fold brighter than a single antibody at early disease stage, increasing to about 150‐fold and 50‐fold brighter in the MTG and SOM, respectively by Braak stage 6.

Overall, this reinforces the hypothesis that at early Braak stages there are a small number of tau aggregates which contain AT8 and T181 phosphorylated tau monomers simultaneously, and that the fraction of these multi‐phosphorylated aggregates and their extent of phosphorylation is increased at later Braak stages.

### 2.5 Super‐Resolution Imaging Reveals a Heterogeneous Tau Aggregate Population With Late Emergence of Long Fibrillar Species

For more detailed measurements of morphology beyond the diffraction limit, tau aggregates were imaged using DNA‐PAINT super‐resolution microscopy [16]. This approach enables nanometer‐scale localization of fluorophore‐labelled antibodies through transient binding events, allowing accurate reconstruction of aggregate size and shape at single‐aggregate resolution provided a sufficient number of binding events are detected. Therefore, small aggregates with few accessible epitopes may not have been imaged in these experiments.

DNA‐PAINT imaging revealed a broad distribution of tau and phosphorylated tau aggregate morphologies across all Braak stages (Figure 4 and Figure S3). In early stages (0–3), most aggregates were small and globular, with mean lengths of 90–100 nm. From stage 5 onwards, the average length increased to ∼120–180 nm in the MTG (Wilcoxon rank‐sum test, Braak stages 0–2 vs. Braak stages 5–6, *p* = 0.009 for AT8 and *p* = 0.009 for HT7), whereas no significant change was observed in the SOM (Figure S3A). This increase did not reflect a uniform elongation of all aggregates, but rather an increase in the proportion of long, fibrillar aggregates, while many aggregates remained short and globular.

Cumulative frequency analysis of aggregate length confirmed that the majority of tau aggregates were short across all disease stages, with a rightward shift of the distribution at late Braak stages (Figure 4B). Similarly, cumulative eccentricity plots showed that most aggregates exhibited low eccentricity values (<0.9), indicating predominantly non‐fibrillar morphologies throughout Braak stages (Figure 4C).

Applying established morphological criteria for long, fibrillar aggregates (>250 nm length and eccentricity > 0.9), we identified a small but significant increase in the proportion of long, fibril‐like aggregates at Braak stages 5–6 in the MTG for both HT7‐ and AT8‐positive tau aggregates (Wilcoxon rank‐sum test, Braak stages 0–2 vs. Braak stages 5–6, *p* = 0.009 and *p* = 0.009, respectively; Figure 4D, Figure S4A,B). These long, fibrillar species remained a minority of the total aggregate population, accounting for approximately 7% of the tau aggregates, by number, at late disease stages. This proportion is based on morphology, not structure, and refers specifically to the diffusible aggregate fraction present in the brain homogenate and detected by our assay, which does not include large non‐diffusible fibrils. In the SOM, a similar trend was observed but did not reach statistical significance.

### 2.6 Emergence of Long Fibrillar Aggregates Implies Increased Contribution From Diseased Subpopulation of Cells Rather Than Altered Growth Kinetics in all Cells

**FIGURE 5:**
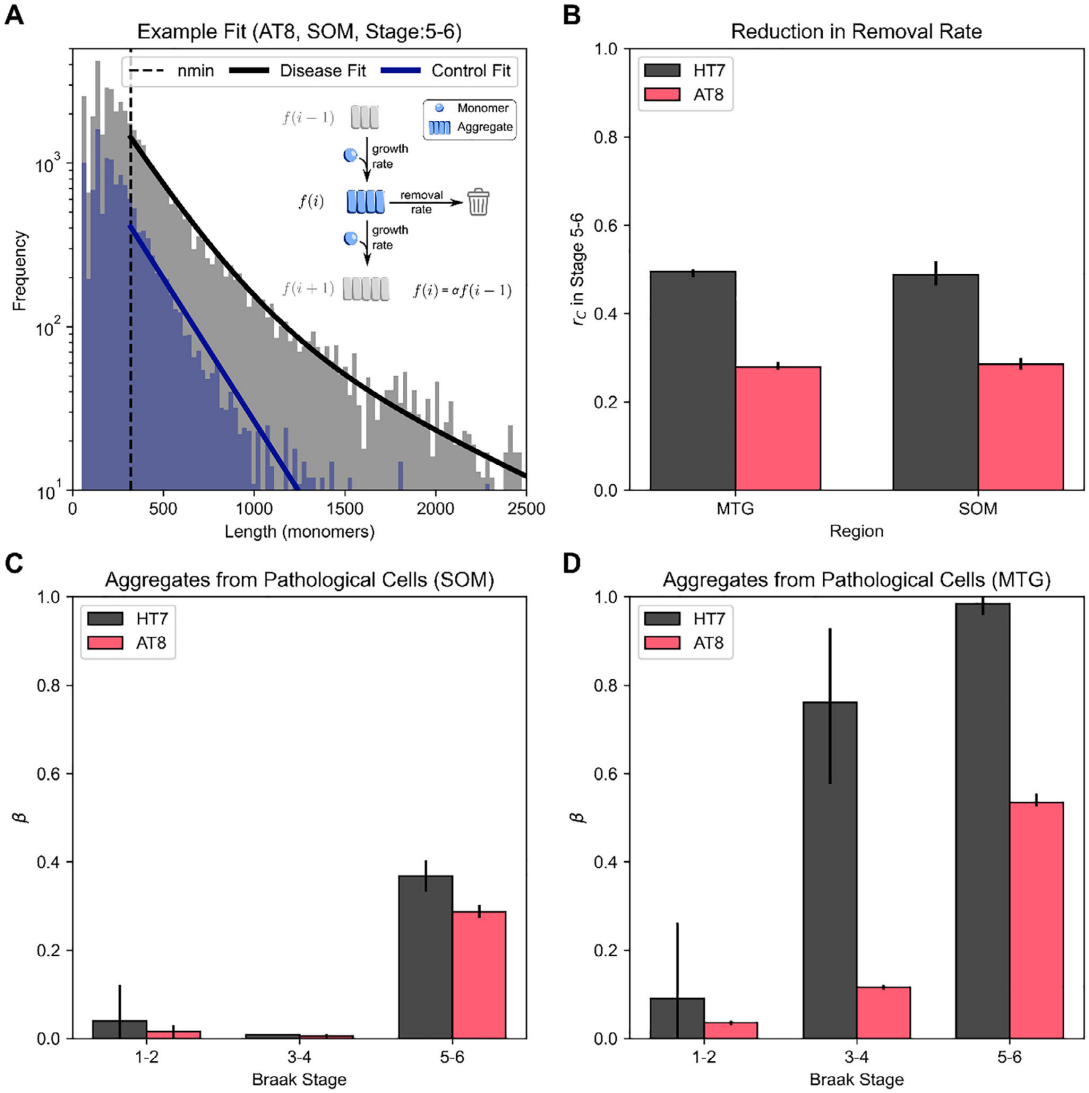
A mathematical model quantifies the difference between health and disease. (A) Exemplar histograms of the different aggregate length distributions observed in health and disease. These observed distributions agree well with the fits from the mathematical model, and the inset shows a schematic of the model. Results from fitting the distributions at different stages of the disease are shown in (B), (C) and (D). The bar height shows the mean value of the posterior and the error bars show the 68% confidence interval. (b) shows the reduction in relative removal in Braak stage 5–6 which varies slightly for each antibody but is conserved well between brain regions. (C) and (D) show how the proportion of fitted aggregates expected to be from pathological cells depends on disease stage.

To explore how this data may elucidate the mechanism of human tau aggregation, we used a model we developed to quantitatively describe the observed aggregate length distribution. We achieved this by modelling the balance of processes that increase the concentration of aggregates of a given size and processes that remove aggregates, see Figure 5A [17]. We built a minimal model by including only two very basic processes known to affect aggregate formation in cells, namely their growth by monomer addition [18] and removal of the aggregate by cellular removal processes, such as autophagy [19]. We only consider aggregates that contain dozens of monomers, so we neglect nucleation processes. To build the simplest possible model, we assume both of these processes, growth and removal, are size‐independent. This minimal model allows us to derive the aggregate size distribution: it is expected to follow a geometric decay *P_n_∼α^n^*, where an aggregate of size *n* occurs with frequency *P_n_* and the factor *α* is related to the ratio of aggregate removal compared to growth, which we call the relative removal rate [17]. Therefore, this relative removal rate can be altered either by a decrease of the absolute removal rate or by an increase of the growth rate.

We predict cells are in either a healthy or pathological state. In the pathological state, the relative removal rate is lower by a factor *r_c_*, resulting in larger *α* and more larger aggregates compared to the healthy state. The prediction of this simple model is that size distributions should follow a straight line when plotted on a logarithmic frequency axis. By contrast, more complex models with size‐dependent removal or growth processes would not generally predict a straight line. Moreover, it is worth noting that changes in the nucleation processes do not affect the distributions, as shown in Cotton et al. [20].

In healthy brains, we expect most cells to be in a stable healthy state. The length distribution of a healthy sample should thus be governed by the average rates of aggregate growth and removal in healthy cells. By contrast, a substantial fraction of cells will be in a pathological state in people with Alzheimer's disease or its prodrome. Healthy and diseased cells may have different relative removal rates, thus, the aggregates in healthy cells follow one geometric distribution, while the aggregates in diseased cells follow another geometric distribution. The size distribution seen in a diseased individual is then a mix of contributions from healthy and diseased cells, giving a sum of two geometric distributions. Indeed, this describes the data from both healthy and diseased individuals well, see Figure 5A. Note that the y axis is Figure 5A is logarithmic. Using a Bayesian approach to fit these distributions (see methods for details), we can estimate the relative contributions of each cell type and the fraction of fitted aggregates that are from pathological cells, *β*. This analysis shows that the contribution of pathological cells to the length distribution begins at Braak stage 3–4 and increases at Braak stage 5–6 for the MTG, but this contribution is lower for the SOM, suggesting that the SOM lags behind the MTG. However, our estimated reduction in the *relative* removal rate between healthy and diseased cells is similar for both brain regions, approximately 30%–50%. Note that this reduction in relative removal could result from either a reduction of the rate of removal or an increase of the rate of aggregation. The longer aggregates that we observed at Braak stage 5–6 can be explained by the higher proportion of aggregates from diseased cells at late stages in this model.

Overall, our data showed a similar size and shape distribution of tau aggregates at all Braak stages. Even at Braak stage 5–6 only a fraction of the tau aggregates were long fibril‐like aggregates. Taken all the data together, we observed increased numbers of more multi‐phosphorylated tau aggregates at late Braak stages, with a similar size and shape distribution.

### 2.7 Weighted Gene Co‐Expression Network Analysis Identifies Pathways Associated With Pathological Tau Aggregate Increase

To gain mechanistic insights into transcriptional changes associated with these aggregate dynamics, bulk RNA sequencing (bulkRNAseq) was performed using the same set of brain samples (Table S3). To identify genes with altered expression associated with tau pathology, we performed differential gene expression analysis comparing AD (Braak stage ≥ 3) samples with Control (Braak stage < 3) samples. 45 genes were upregulated with AD, while 2 genes showed downregulation. Top upregulated transcripts included immune response and inflammation‐related genes *CX3CR1, HLA‐DRB5, IGSF6*, and *P2RY12* (Figure S5A and Table S4). To understand the functional relevance of these genes, we performed pathway enrichment analysis based on the Gene Ontology (GO) Biological Process and Wiki pathways database. Enriched terms included microglial migration, TYROBP causal network in microglia, antigen processing and presentation, and inflammatory response pathways (Figure S5B and Table S5), supporting the well‐established role of increased microglial activation and inflammation in AD [21].

**FIGURE 6:**
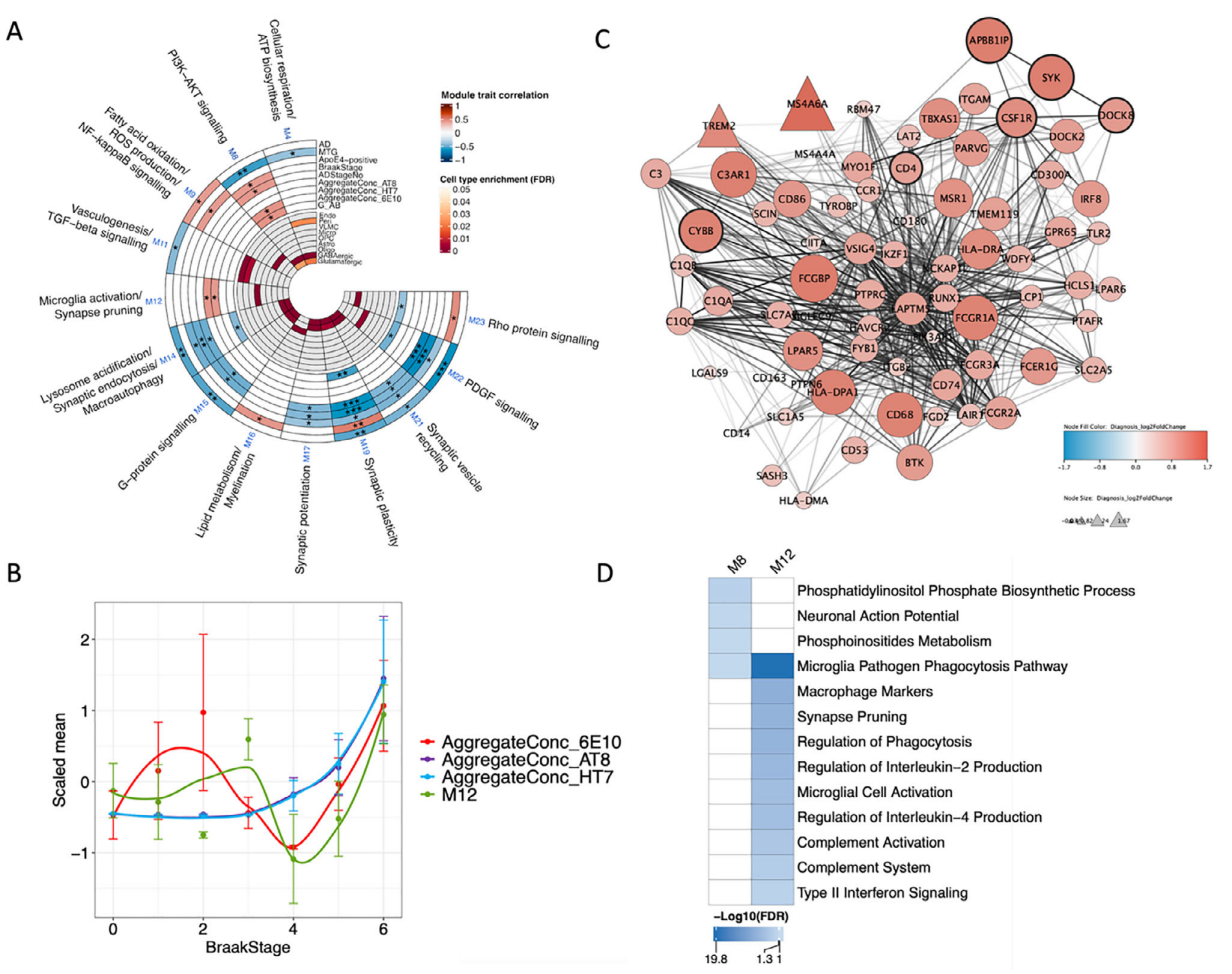
Weighted gene co‐expression network identifies gene modules associated with tau pathology. (A) Circos plot visualizing module‐trait correlations (Pearson's) highlighting associations with key AD features (positive association in red, negative in blue). Student asymptotic *p*‐value was calculated for Pearson's correlations (^*^ *p* ≤ 0.05, ^**^ *p* ≤ 0.01, ^***^ *p* ≤ 0.001) across all 25 samples. However, these p‐values do not account for intra‐donor correlation between the two (regional) samples from each donor. Region‐stratified correlations, which remove this confound, are provided in Figure S6 and are the basis for the more detailed reporting of associations described in the text. Cell type enrichment in the inner circle indicates significant enrichment in red (FDR ≤ 0.05) and NS in white. MTG, middle temporal gyrus; G_AB, 4G8 positive amyloid‐beta immunostained area; FDR, false‐discovery rate; Astro, astrocytes; Micro, microglia, OPC, oligodendrocyte progenitor cell, Oligo, oligodendrocyte, VLMC, vascular leptomeningeal cell; Endo, endothelial cells; Peri, pericytes. (B) Scaled mean displaying distribution of module eigengene and aggregate concentration of different AD associated pathology against Braak stages. Filled dots represent means across samples and error bars denote standard error. (C) Network plot highlighting genes from M12. The fill color represents the log2 fold‐change (FC) of differential expression of the genes between AD (Braak ≥ 3) and non‐disease healthy Control (Braak < 3) individuals. The thicker black border represents significantly differentially expressed genes at FDR ≤ 0.05. Genes identified as causal for AD risk are highlighted in triangles [23]. The edge color represents the strength of the pairwise correlations between the genes. (D) Top enriched pathways from modules M8 and M12 associated with amyloid‐beta and tau aggregate quantities, respectively. Fill color showing ‐log10 transformed FDR of the significance *p*‐value.

Gene network analysis provides insights into biological function by identifying modules of co‐expressed genes that are likely to be involved in shared biological processes. To identify gene modules associated with disease traits, we used weighted gene co‐expression network analysis (WGCNA) [22], which identified 28 gene modules (M1–M27, Figure 6A and Table S6, M0 consists of a group of unassigned genes). Network construction used all 25 samples utilizing two regions per donor by design, as MTG and SOM differ substantially in pathological burden and together provide the range of variation required for co‐expression network inference. To prioritize functionally relevant modules, module eigengenes (the first principal component (PC) of module gene expression) were correlated with aggregate measurements, neuropathological features, Braak stage or other relevant descriptors (e.g. Sex). The two regional samples from each donor are not statistically independent, therefore, these correlations were computed within each region separately, so that every donor contributes a single observation (Figure 6A, Figure S6A–C and Tables S7 and S8). The M8 eigengene was positively correlated with Braak stage, amyloid‐beta aggregate quantity (6E10), and 4G8‐positive amyloid‐beta immunostained area in grey matter (G_AB) in both regions analyzed separately, with associations reaching significance in SOM (Braak stage, *r* = 0.73, *p* = 0.005 and G_AB, *r* = 0.56, *p* = 0.047; *n* = 13) and showing consistent positive direction in MTG. This suggests that these genes are associated with responses to amyloid‐beta aggregation in AD. Since we were interested in what drives tau aggregation, we next examined M12, whose eigengene expression was related to both total tau (AggregateConc_HT7) and p‐tau (AggregateConc_AT8) aggregates measured using Simoa. These associations were moderate in MTG (AggregateConc_HT7, *r* = 0.46 and AggregateConc_AT8, 0.49; *n* = 12) and weaker in SOM (*r* = 0.22 for both; *n* = 13). The correlations did not reach significance in either region separately. Notably, in contrast to M8, M12 showed little association with amyloid‐beta measures in either region, indicating that the two modules track distinct aspects of pathology (Figure 6A, Figure S6A–C).

To assess how robustly these correlations were estimated in a cohort of this size, we recomputed every module–trait correlation within each region omitting one donor at a time and obtained 95% confidence intervals by bootstrap resampling of donors. Stability was positively associated with effect size: associations with |r| > 0.4 were almost uniformly consistent in direction across all leave‐one‐donor‐out fits, whereas weaker associations frequently changed sign, as expected where the estimate lies close to zero (Figure S7A). The M8 associations in SOM were the most robust in the dataset, remaining directionally stable across every fit with confidence intervals excluding zero. We therefore interpret module–trait associations as indicative of direction rather than as precisely estimated effect sizes. Given the cohort size, we assessed the robustness of module construction directly. Module structure was preserved in an independent MTG cohort (*n* = 38), with all 27 modules showing evidence of preservation and ranking above randomly sampled (gold module) and unassigned reference gene sets (grey module) (Figure S7B).

To better define the relative expression of M12 transcriptional and aggregate abundance changes at different stages of disease, we represented module expression as a function of Braak stage across the sample set (Figure 6B, Figure S8). In this Braak “pseudo‐time,” M12 expression followed a similar but delayed trend relative to amyloid‐beta aggregate concentration, suggesting that M12 activation is downstream of amyloid‐beta pathology but upstream of tau pathology. M12 genes showed a significant (FDR 2.6e‐10) overlap with the up‐regulated genes (*CD4, APBB1IP, DOCK8, SYK, CYBB, CSF1R*) detected in AD compared to Control samples (Figure 6C). Other notable genes in M12 are AD GWAS genes involved in immune cell activation (*TREM2, MS4A4A, MS4A6A*) and genes related to complement cascade (*C3AR1, C3, C1QA, C1QB, C1QC*) (Figure 6C).

To explore potential cellular contributions to the co‐expression modules, we conducted cell‐type enrichment analysis of the modules using expression‐weighted cell type enrichment (EWCE) [24], against cell‐specific gene expression signatures derived from those of single‐nuclear transcriptomes in the Allen Human Brain Atlas [25]. M12 showed enrichment for genes expressed in microglia (Figure 6A and Table S9).

Pathway enrichment analyses suggested functional roles for proteins encoded by the genes in these modules (Table S10). M12 was enriched for pathways related to microglial activation, including those involved in synapse pruning (*C1QB, C3, ITGAM, TREM2, C1QC*), regulation of chemokine production (*CSF1R, CD74, IL18, TREM2, LCP1, TLR2*), and response to low‐density lipoprotein particle stimuli (*FCER1G, ITGB2, TREM2, LCP1, CD68*). From these data, we hypothesize an association between pro‐inflammatory microglial activation and tau aggregate accumulation (Figure 6D). Individual genes making larger contributions to the M12 eigengene included some that have been associated with complement response (*C3AR1, C3, C1QB, C1QC*), microglial activation and phagocytosis (*MSR1, CD68, FCGR2A*) (Figure S9A,B). M8 contained genes previously associated with phosphatidylinositol signaling (*PIK3CA, PIK3CB, PIP5K1B*), negative regulation of protein depolymerization (*PIK3CA, CKAP2, TTBK2*) and MAP kinase activity (*FLT3, KIT, DKK1*), and with GABAergic and glutamatergic neurons (Figure 6A,D). M8 eigengene expression increased with tissue‐level amyloid‐beta immunostaining. We hypothesize that this reflects an association between neuronal stress and increased aggregate load (Figure S8). Modules negatively correlated with AD or increasing Braak stage (e.g. M14, M15, M17, M19, M21, and M22), were enriched for genes expressed in neuronal transcriptional pathways for autophagy and protein folding, metabolic signaling and synaptic signaling (Figure 6A and Figure S11). The inverse correlations provide evidence for potentially maladaptive downregulation of these homeostatic pathways at later stages of AD.

### 2.8 Endo‐Lysosomal Dysfunction and Complement Cascade Activation in Microglia Are Associated With Increases in Tau Aggregates

**FIGURE 7:**
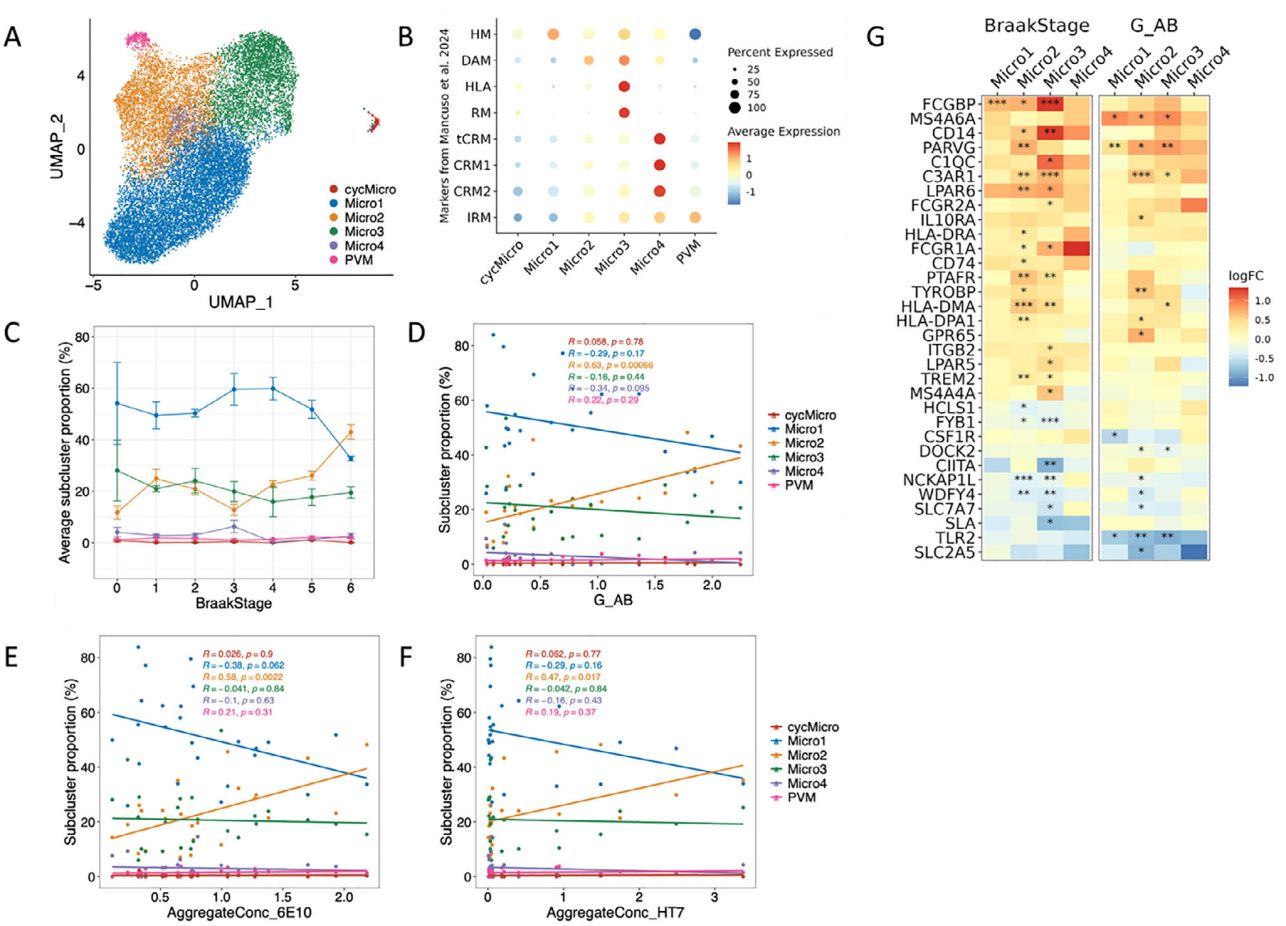
snRNAseq analysis identifies inflammatory microglia sub‐population associated with aggregate quantities. (A) UMAP (Uniform Manifold Approximation and Projection) visualization of microglial subpopulations in snRNAseq. (B) Dotplot showing percentage of cells expressing the average scaled expression of marker geneset [27] for each microglia sub‐cluster. cycMicro, proliferating microglia; PVM, perivascular macrophage. (C) Average proportion of microglia subclusters against Braak stages. Error bars denote standard error. (D–F) Correlations of microglia subclusters with grey‐matter amyloid‐beta (G_AB) immunostained area [D], the Simoa‐derived concentration of 6E10 (amyloid‐beta) [E], and total tau (HT7) aggregates [F]. The X‐axis shows scaled but non‐centered aggregate concentrations. (G) Heatmap visualizing the differential expression of M12 genes in snRNAseq data, referring to genes initially identified from bulk RNA seq, in regression analysis against Braak stages and the grey‐matter amyloid‐beta immunostained area (G_AB). Genes considered as differentially expressed with an absolute log fold‐change logFC ≥ 0.25 and FDR ≤ 0.05 (*FDR≤0.05, **FDR≤0.01, ***FDR≤0.001).

Amyloid‐beta‐associated microglial inflammation implicates complement activation in downstream tau pathology. To further confirm glial transcriptional changes linked to tau aggregate formation as observed in our bulkRNAseq dataset, we used fluorescence‐activated sorting (FACS) [26] to enrich for microglial and astrocyte nuclei by depleting neuronal and oligodendrocyte nuclei from the same cohort (Table S11). Single nuclear RNA sequencing (snRNAseq) of this enriched population allowed the selective analysis of transcripts in nuclei from microglia and astrocytes. We then sub‐clustered these populations to define nuclei associated with specific functional sub‐clusters (states) of these cells (Figure 7A and Tables S12 and S13). We found that the proportion of the Micro2 sub‐cluster, which expresses gene signatures expressed in disease‐associated microglia (DAM) [27], was higher in nuclei in samples from brains with Braak stage 3 or higher pathology (Figure 7A–C). We found that the proportion of microglia in brains that were found in the Micro2 sub‐cluster correlated positively with both the amyloid‐beta immunostaining and with the amounts of tau aggregates (Figure 7D–F).

To integrate the gene modules identified in bulkRNAseq with our snRNAseq data, we compared the module genes with the differentially expressed genes in snRNAseq, specifically in M12 (Table S14). Several M12 genes were differentially expressed in the Micro2 and Micro3 sub‐clusters in association with greater amyloid‐beta area or Braak stage (Figure 7G). These included genes encoding proteins of the complement pathway (C1QC, C3AR1) and those associated immune signaling (FCGBP, MS4A4A, MS4A6A, FCGR2A, FCGR1A), antigen presentation (HLA‐DPA1, HLA‐DMA, HLA‐DRA, CD74), and TREM2 signaling (TREM2, TYROBP). As increased proportions of Micro2 sub‐clusters are associated with higher Braak stages and M12 eigengene expression follows a similar but delayed trend to that seen with amounts of amyloid‐beta aggregates, we hypothesized that these pro‐inflammatory microglia arise as a response to amyloid‐beta accumulation and then directly or indirectly regulate tau aggregation.

To investigate whether amyloid‐beta aggregates influence tau aggregation through gene expression, we performed mediation modelling within the MTG dataset, which showed a stronger amyloid–tangle association than did that from the SOM (rho = 0.89, Bonferroni‐adjusted *p* = 1.2 × 10^−^^4^, Figure S10A). Age at death was retained as a prespecified confounder. The results of this analysis are only able to be used for hypothesis‐generation as none of the individual gene transcripts survived FDR correction. Transcripts were prioritized by the average causal mediation effect (ACME), the change in tangle load, in standard deviation units, attributable to a one standard deviation increase in amyloid‐beta acting through gene expression (i.e., mediator). Four genes showed indirect effects whose 95% confidence intervals excluded zero: *BCYRN1* (ACME = −0.38), *PLEKHA2* (ACME = −0.23), *RECK* (ACME = −0.37) and *VPS36* (ACME = −0.24). Among the next most highly ranked candidates, with intervals marginally overlapping zero, were *GRN* (ACME = 0.34), *GYPC* (ACME = 0.32), C3AR1 (ACME = 0.31), and *PLD3* (ACME = 0.24) (Figure S10B). Some of the encoded proteins have endolysosomal functions, including ESCRT‐dependent endosomal sorting (VPS36), a lysosomal protein implicated in late‐onset Alzheimer's disease risk (PLD3), and lysosomal biogenesis and secretion (GRN). *C3AR1*, a complement receptor and member of the microglial M12 module, ranked ninth of 5366 genes tested.

To further test the microglial component of this signal with greater power, we utilized a larger independent snRNAseq dataset [28], in which two microglial clusters, Mic.12 and Mic.13, were associated with amyloid‐beta and neurofibrillary tangle immunostaining and increased in expression from Braak stage 2–3 (Figures S12–S14). We restricted mediation analysis to the 76 M12 genes represented in that dataset, allowing false discovery rate correction within a hypothesis‐driven gene set rather than across the entire transcriptome. M12 was defined by co‐expression in the bulkRNAseq data presented in the current study, without reference to pathology or to any mediation result, therefore restricting the mediation analysis to M12 gene in the independent cohort does not result in circularity.

In Mic.12 (*n* = 415 donors), 21 of 76 M12 transcripts showed evidence of mediating the amyloid–tangle relationship at FDR < 0.1, top ranked (by adjusted p‐value) mediator transcripts included *C3AR1* (adjusted *p* = 0.051), *HLA‐DPA1, SLA, ITGB2, HCLS1* and *LAPTM5*, together with further complement, antigen‐presentation and microglial activation gene expression from, e.g., *C1QC, CSF1R, TYROBP, TMEM119* and *CD68* (Figure S15A, Table S15). Individual transcripts accounted for a small proportion of the total amyloid–tangle effect (3%–8%), consistent with distributed rather than single‐gene mediation. No transcript reached FDR < 0.05, and none of the M12 transcripts showed evidence of mediation in Mic.13 (*n* = 337), suggesting that this signal is specific to the Mic.12 population.

Given the sample size of the discovery cohort and the absence of FDR‐significant findings there, we interpret the discovery estimates as indicative of direction rather than magnitude and regard these transcripts as candidates for targeted testing rather than established mediators. The M12‐restricted analysis in the larger cohort provides additional support for a model in which amyloid‐beta‐driven microglial activation, involving complement and antigen‐presentation programmes, contributes to tau aggregation.

### 2.9 Neuronal Subpopulations Differ in Transcriptional Response and Vulnerability to Tau Aggregation

**FIGURE 8:**
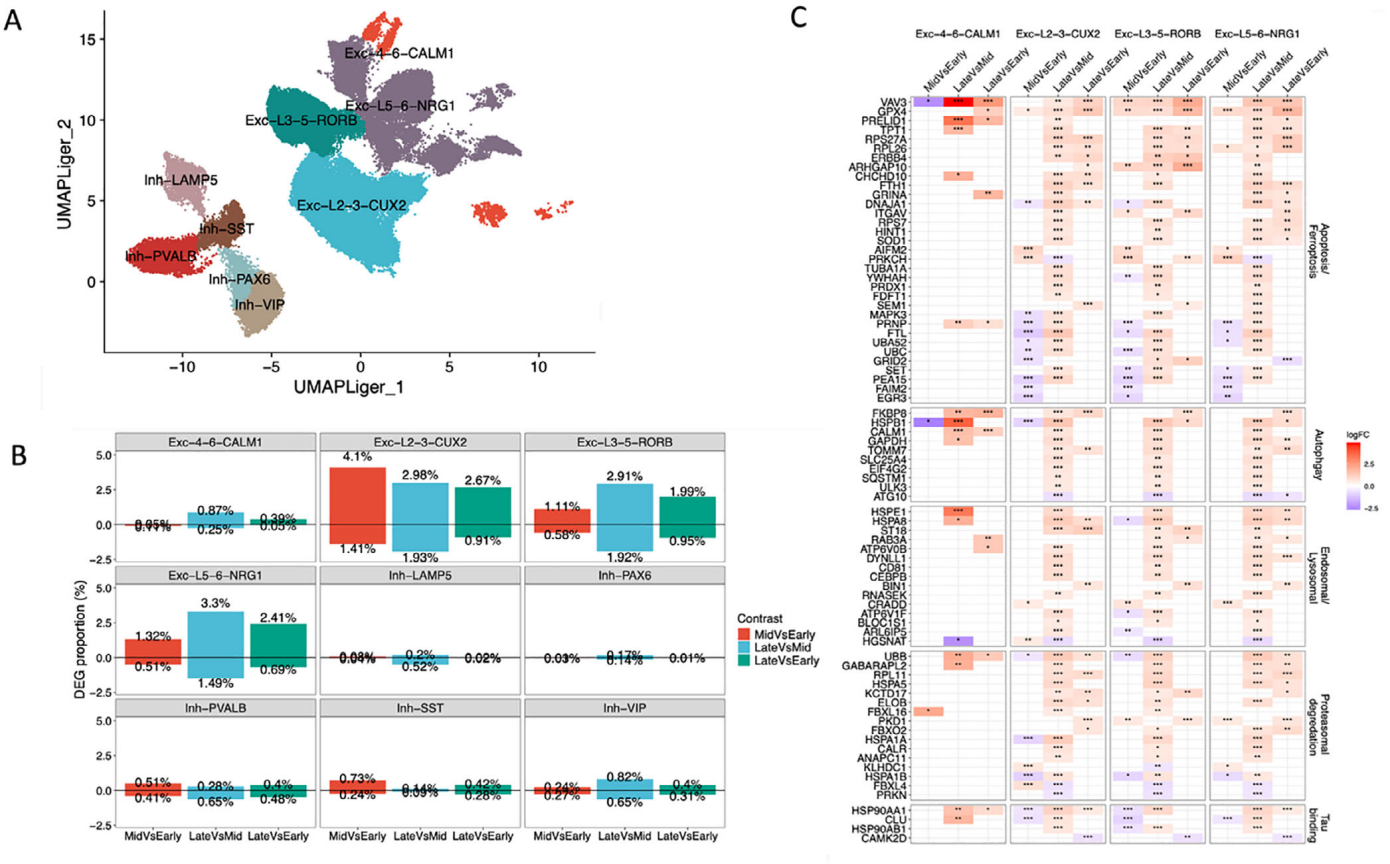
snRNAseq analysis revealed neuronal response with increased aggregate quantities. (A) UMAP visualization of major neuronal sub‐populations in snRNAseq data. (B) Bar plot showing the proportion of genes differentially expressed in each neuronal subpopulation. Differential expression was performed comparing mid (Braak 3–4) and late (Braak 5–6) AD samples with Early (Braak 0–2) samples within each of the sub‐populations. (C) Heatmap showing significantly differentially up‐ or down‐regulated genes in samples with mid (Braak 3–4) and late (Braak 5–6) AD compared to those of early (Braak 0–2) samples in all excitatory neuronal sub‐populations. Genes considered as differentially expressed with an absolute log fold change (logFC) ≥0.5 and FDR ≤ 0.05. Genes are grouped based on their known functional association with gene ontology (GO) terms.

Finally, to examine neuronal transcriptional changes that contribute to or respond to tau aggregation, we profiled all cell types from the same brain samples using snRNAseq and identified diverse excitatory and inhibitory neuronal sub‐populations [25] (Figure 8A and Table S16).

To assess gene expression changes with the progression of pathology, we performed differential expression comparing mid (Braak 3–4) and late (Braak 5–6) AD samples with Early (Braak 0–2) samples (Table 18). Overall, excitatory neurons showed enhanced response at all Braak stages (Figure 8B). The Exc‐L2‐3‐CUX2 sub‐population showed the most changes in gene expression at mid stage (Braak 3–4) compared to the early stage (Braak 0–2) (Figure 8B). Three neuronal sub‐populations (Exc‐L2‐3‐CUX2, Exc‐L3‐5‐RORB and Exc‐L5‐6‐NRG1) showed consistent gene expression changes at different disease stages, suggesting a conserved molecular response to pathology (Figure 8C and Figure S16). We did not find evidence for increases in MAPT (encodes tau protein) gene expression at later stages of AD. This suggests that increased tau aggregation is unlikely to arise from increased tau transcription, implicating post‐transcriptional or clearance‐related mechanisms instead. Notable differentially upregulated genes included tau‐binding proteins *HSP90AA1, HSP90AB1* and *CLU* (Figure 8C). HSP90AA1, a molecular chaperone, is known to interact with tau to promote the formation of toxic oligomer species [29], while CLU (encodes clusterin) has recently been found to promote tau spread [30]. On the contrary, *CAMK2D*, encoding a Ca2+/calmodulin‐dependent protein kinase II, known to potentiate tau phosphorylation [31] was downregulated at the late stage (Figure 8C). Pathway enrichment analysis of differentially expressed genes revealed the enrichment of cellular signaling pathways (Figure S17, MidvsEarly) in the initially upregulated genes, followed by a broader range of pathways being enriched at later stages (Figure S17 LateVsMid and LateVsEarly). Neuronal sub‐populations (Exc‐L2‐3‐CUX2 and Exc‐L3‐5‐RORB) displayed the enrichment of downregulated transcripts for those encoding proteins with potential roles in aggregate clearance and the response to unfolded proteins (Figure S18 MidVsEarly) many of which were upregulated at late stages (Figure S17 LatevsMid and LatevsEarly). Upregulated pathways at late stages were enriched for those associated functionally with Tau binding, autophagy, cellular bioenergetics and neuronal apoptosis (Figure 8C, Figure S17) suggesting a later Braak stage compensatory mechanism response to the AD pathology.

To understand how different neuronal sub‐populations contribute to the increase in tau aggregate length, we correlated the proportion of each neuronal subtype with the mean fraction of total tau aggregates derived from diseased neuron (denoted as MeanBeta_HT7) (Figure S19). MeanBeta_HT7, calculated from the tau aggregate length distribution model described in Figure 5. serves as a proxy for the fraction of diseased neuron in each sample. The Exc‐L2‐3‐CUX2 showed a decrease in proportion with increasing MeanBeta_HT7, suggesting a relative vulnerability of this population to tau pathology. In contrast, Exc‐L5‐6‐NRG1 and Inh‐LAMP5 proportions were significantly positively correlated with MeanBeta_HT7, indicating a greater contribution of these neurons to the overall increase in total tau aggregate length.

Together, our findings suggest that neuronal sub‐populations exhibit different transcriptional responses to pathology at different stages of AD. We hypothesize that the increase in neuronal expression of transcripts associated with protein clearance pathways with higher Braak stages may reflect intrinsic resilience mechanisms that could delay neurodegeneration.

## 3 Discussion

We have studied the tau aggregates that form in two brain regions during the development of AD in detail. The immunohistochemistry showed, as expected, increased amyloid deposition followed by a rapid increase in the amount of tau deposited at late Braak stages (Figure 2A). We then characterized the nanoscopic tau aggregates, including smaller diffusible species, in the brain homogenate using single‐molecule methods. With Simoa and SiMPull we showed tau aggregates are present even in non‐dementia donors at Braak stage 0, albeit at low levels, and a marked increase in number at late Braak stages (Figures 2B and 3B). Colocalization experiments showed that multi‐phosphorylated tau aggregates formed that increased in quantity with disease progression, suggesting multi‐site phosphorylation plays an important role in tau aggregation at later Braak stages (Figure 3C). Super‐resolution imaging of the tau aggregates revealed that they were heterogeneous in size and shape, but similar at all Braak stages until late Braak stage, when more long aggregates formed (Figure 4B–D). These data show that, while morphologically fibril‐like aggregates form, they are not the dominant tau species, even at late‐stage AD, less than 10% of the aggregates. These results are in agreement with the differences in the frontal cortex that we previously observed between Braak stage 6 AD brains and control brains [5] and preceding work [6, 32]. Recent work imaging in fixed brains confirms these findings [33]. However, the morphological classification by super‐resolution microscopy ideally would need to be confirmed by direct structural measures, e.g., with cryo‐electron microscopy.

Our analysis of the size distribution of the tau aggregates provides mechanistic insights. It shows that the increase in tau aggregation occurs early in disease in MTG, related to the presence of more diseased neurons, with trends towards increased aggregate quantity from Braak stage 3 becoming significant in Braak stages 5–6. The increase in aggregate length observed beyond Braak stage 5 is explained by an increased contribution of tau aggregates from diseased cells. Given that the relative removal rates in the diseased and healthy populations remain constant, there is no evidence for a change in aggregation mechanism within the healthy or diseased cells at later Braak stages. Interestingly, although the Braak stage in which neurons express pathology is different between the MTG and SOM, when the neurons become diseased, the relative aggregate removal rate is estimated to be reduced by 50% for HT7 tau aggregates in both brain regions. Overall, this analysis suggests that similar aggregation and removal processes occur in the MTG and SOM, but that pathology is less advanced in the SOM.

Our integrative analysis of the aggregates and transcriptomic data generated from the same set of brain tissues allowed us to directly map transcriptional changes to disease progression stages. Our analysis provides insights into the processes that occurred concurrently with increased amyloid‐beta or tau aggregation. We showed that microglial inflammation and complement activation‐related gene module positively correlates with tau aggregation. Furthermore, the proportion of disease‐associated microglia also positively correlated with increased tau aggregates. Finally, our mediation analysis outcome is in line with previous findings where C3a receptor (C3AR1) was shown to be a critical regulator of tau pathology. Based on this, we hypothesize a model in which amyloid‐beta deposition is an early event that precedes microglial inflammation and complement activation, which may in turn drive tau aggregation [34]. Our results further complement previous findings that demonstrated that microglial activation and tau propagation co‐occur in a Braak‐like pattern and that amyloid‐beta potentiates the effect of microglial activation on tau spreading [35]. Furthermore, we observed no increase in tau expression with Braak stage, emphasizing the importance of studying aggregates and the mechanisms contributing to their aggregation and removal. We observed an AD‐associated decline in relative amounts of those neurons that fail to trigger adaptive clearance responses to pathology (Figure 8B). In contrast, neurons expressing increased protein clearance‐associated genes even with low pathology exhibit resilience (Figures S17 and S18). Finally, we showed that neuronal sub‐population displaying relative resilience to cell death contributes to the increase in total tau aggregate length uncoupling the tau aggregate generation and neuronal death susceptibility. Overall, our study suggests that increased inflammation and a lack of protein aggregate clearance may be major drivers of tau aggregation although this conclusion is based on associations that correlations cannot be proven causal in the context of a study limited to observations postmortem. This hypothesis and other supporting data are discussed in the next section.

### 3.1 A Hypothesis for the Mechanism of Regional Increase in Tau Aggregation

Our transcriptomics data suggest that the mechanism leading to increased tau aggregation appears mediated by microglial inflammation and activation of the complement pathway downstream of amyloid‐beta aggregation. Our recent paper supports this hypothesis, showing that exposure to TNF leads to increased tau aggregation in iPSC neurons and extracellular release of tau aggregates [36]. We have also shown that hyperphosphorylated tau aggregates can induce inflammation in human macrophages in vitro [7]. As the literature and our current data suggests, increased phosphorylation would cause tau to detach from the microtubules, raising the tau concentration in the cytosol and hence increasing tau aggregate formation. One neuron may release amyloid‐beta or tau aggregates that trigger microglial inflammation [7] and this inflammation could trigger stress pathways in neurons, leading to tau hyperphosphorylation and consequent aggregation in neighboring neurons, leading to the observed increase in tau aggregates. This seems a plausible mechanism, consistent with each of our experimental observations and literature reporting that TNF is raised early in the AD brain and PET showing concurrent spread of inflammation and tau aggregation in AD patients [37]. Inflammation‐induced cell death, necroptosis, has also been shown to increase with Braak stage in support of this mechanism [38].

One intriguing observation from our study is that a mix of aggregated species, with a continuum in sizes and phosphorylation states, was observed across all disease stages, and there was no single fibril or HMW tau aggregate type that dominated the aggregate population. This is in disagreement with some expressions of prion‐like spreading models that posit a tau seed being released by a neuron, resulting in seeding aggregation and replication of aggregates in a neighboring neuron and the dominance of a single prion‐like species being responsible for propagation and disease progression. Our findings instead imply that propagation of pathology is either mediated by an indirect inflammation mechanism, or if it involves the transfer of seed aggregates, these seeds have to replicate into a diverse population of aggregates, rather than a single pathological species. In agreement with our findings, a recent tauopathy mouse model with FTD‐causing mutations in the humanized MAPT gene showed abundant hyperphosphorylated tau in the hippocampus and entorhinal cortex, neurite degeneration, loss of viable synapses and indicators of behavioral abnormalities, yet no seed‐competent fibrillar structures were detected [39]. This paper shows that fibrillar tau species are not required for tau‐mediated neurodegeneration. Together with our findings on inflammation above, this observation highlights that small tau aggregates and large fibrillar structures may contribute to disease through distinct mechanisms and at different stages of disease.

## 4 Conclusions

We have found that the key changes in the tau aggregates at later stages of AD are their phosphorylation state and number, with more modest changes in size, and that these changes correlate with increased microglial inflammation. Our data suggests the hypothesis of an indirect mechanism whereby microglial inflammation induces tau aggregation in several neighboring neurons leading to more inflammation, spread of tau pathology through the brain and the increase in small tau aggregates at later stages of disease.

## 5 Limitations of the Study

There are several limitations to this work. Firstly, we are deducing the mechanism of neuronal death from measurements on surviving neurons, and our methods do not detect all the tau aggregates present. We have used postmortem brain so have deduced mechanisms using correlations since it is not possible to perform longitudinal experiments per donor. This study looks at two brain regions, with a limited sample size per Braak stage: future studies should look at other brain regions and additional donors, now that the methodology has been established and validated. Whilst our mediation analysis identifies gene expression trends consistent with amyloid‐beta‐driven microglial inflammation contributing to tau aggregation, this analysis cannot establish causality or temporal ordering. Given insufficient statistical power for multiple testing correction, we reported nominal *p*‐values. Therefore, the results should be interpreted as hypothesis‐generating rather than confirmatory.

On their own, our results also do not explain the pseudo‐temporal delay or comparatively limited increase in tau phosphorylation and aggregation in the SOM in AD compared to MTG, observed with our single‐molecule studies and in typical immunohistochemistry Braak staging. It is possible that the SOM may be more resilient to the inflammation that causes the tau aggregation. The MTG has high metabolic activity since it is used in both rest and higher‐order cognitive functions, possibly making this region more vulnerable. More detailed studies with greater sample power are needed to establish what is responsible for the delayed tau aggregation in the SOM, using our approach of combining single‐molecule measurements of amyloid‐beta and tau aggregates with transcriptomics in matched donor samples.

## 6 Methods

### 6.1 Immunohistochemistry (IHC) and Image Analysis

IHC was performed on formalin‐fixed paraffin‐embedded (FFPE) sections from the MTG and SOM of each brain studied and paired with material from the cryopreserved contralateral hemisphere used for all other experiments in this study. After dewaxing and rehydration of slides, endogenous peroxidase activity was blocked with 0.3% H_2_O_2_, followed by antigen retrieval, detecting amyloid‐beta with 4G8 (BioLegend, 800711; 1:15000 dilution), and phosphorylated tau with AT8 (ThermoFisher, MN1020; 1:1600 dilution) or PHF‐1 (The Feinstein Institutes for Medical Research; 1:5000 dilution). Primary antibodies were incubated overnight at 4°C. All stains were applied at pH6 and steamer using Super Sensitive Polymer‐HRP staining kit (Biogenex, QD552‐GPEN) for antibody visualization. Tissue was counter‐stained by incubation in Haemalum Mayer (TCS Biosciences, HS315‐1L) for 2 min, followed by dehydration, clearing and mounting.

Digital images were generated from IHC‐stained slides, scanned using a Leica Aperio AT2 Brightfield Scanner (Leica Biosystems). Images were analyzed using Halo software (Indica Labs) after optimization of Indica Labs macros for each antibody (Multiplex IHC v1.2).

### 6.2 Brain Homogenate Preparation

This homogenization method is used routinely to extract brain proteins into liquid [40]. In summary, 50 mg of fresh‐frozen brain tissue was homogenized in 500 µL of homogenization buffer (10 mM Tris‐HCl, 0.8 M NaCl, 1 mM EGTA, 10% sucrose, 0.1% sarkosyl, complete protease inhibitor tablet (Merck Roche, 4693132001), PhosSTOP phosphatase inhibitor tablet (Merck Roche, 4906845001); pH 7.4) at 4°C at 5 m/s in a VelociRuptor V2 Microtube Homogenizer (Scientific Laboratory Supplies, SLS1401) for two 20 s cycles with a 10 s interval. The homogenate was centrifuged at 4°C at 21 000 x g for 20 min. The upper 90% of the supernatant was extracted and retained, whilst 250 µL of homogenization buffer was added to the pellet for a second round of homogenization and centrifugation. The upper 90% of the resultant supernatant was extracted and combined with the original supernatant, then aliquoted, flash frozen with dry ice and stored at −80°C. Braak staging and brain region information was blinded at the point of homogenization, using randomized labelling, and not unblinded until data analysis, to minimize experimental bias in BCA, SiMPull and STORM experiments.

### 6.3 Total Protein Concentration Quantification

Total protein concentration of brain homogenates was quantified by the Bicinchoninic acid (BCA) Protein Assay Kit (Thermo Scientific Pierce, 23225), following the manufacturer's protocol.

### 6.4 Antibody Preparation

Monoclonal antibodies against human tau (HT7, specific to tau 159–163), phosphorylated tau (AT8, specific to tau phosphorylated at serine 202 and/or threonine 205, and T181, specific to tau phosphorylated at threonine 181) and Aβ (6e10, specific to 1–16 amyloid‐beta and APP) were used in this study.

#### 6.4.1 Antibody Biotinylation

Biotinylated antibodies were required to bind the passivated single‐molecule pull‐down (SiMPull) surface for protein capture, and to bind SBG for protein detection in Simoa. Biotin‐conjugated HT7 (Invitrogen, MN1000B), AT8 (Invitrogen, MN1020B) and 6E10 (BioLegend, 9340‐02) were commercially available. T181 (abcam, ab236458) was biotinylated using the Biotin Conjugation Kit (Fast, Type B)—Lightning‐Link (abcam, ab201796) following manufacturer's instructions. Excess biotin was removed using a 40 kDa Zeba Spin desalting column (Thermo Fisher, A57756) followed by a 50 kDa Amicon Ultra Spin column (Merck, UFC5050). The biotin‐conjugated antibody was stored at 4°C. The protein concentration was determined through absorption at 280 nm.

#### 6.4.2 Antibody Dye Labelling

Antibodies were labelled with fluorophores for protein aggregate detection in microscopy experiments. HT7 and AT8 antibodies (Thermo, MN1000 and MN1020) were labelled with Alexa Fluor 647 using the Alexa Fluor 488 Conjugation Kit (Fast)—Lightning‐Link (Abcam, ab269823) as per manufacturer's instructions. T181 (abcam, ab236458) antibody was labelled with Alexa Fluor 488 using the Alexa Fluor 488 Conjugation Kit (Fast)—Lightning‐Link (abcam, ab236553) as per manufacturer's instructions. The labelled antibodies were cleaned up using a 0.5 mL 40 kDa MWCO Zeba Spin Desalting Column followed by Amicon buffer exchange to PBS until clear flowthrough. The protein concentration and degree of labelling were determined through absorption at 280 and 494 nm (AF 488) or 650 nm (AF647), taking into account the absorption of the dyes at 280 nm. The degree of labelling was within the manufacturer's recommendation of 3–9 moles of dye per mole of antibody.

### 6.5 Single Molecule Array (Simoa)

#### 6.5.1 Bead Conjugation

Conjugation of antibodies onto Simoa 488‐dye labelled paramagnetic carboxylated beads (Quanterix, 103612) was performed using EDC chemistry according to the manufacturer's instructions and as per published work [10]. The 0.3 mL of beads at 1.4 × 10^9^ beads/ml stock concentration was washed three times with the Bead Wash Buffer (Quanterix, 101355), then twice with Bead Conjugation Buffer (Quanterix, 101357). The beads were activated in ice‐cold 0.3 mg/mL EDC (Quanterix, 100022) for 30 min at 4°C on a rotator before washing again with ice‐cold Bead Conjugation Buffer. In the meantime, 100 µg of antibody underwent buffer exchange to Bead Conjugation Buffer with a 50 kDa Ultra Centrifugal Filter (Merck Millipore Amicon, UFC505008). The antibody concentration was determined by A280 Nanodrop spectrophotometer. The beads were resuspended in 0.2 mg/mL antibody in 300 µL Bead Conjugation Buffer for 2 h on a rotator at 4°C. Excess antibody was removed by washing twice with Bead Wash Buffer. Antibody coating efficiency could be determined from A280 Nanodrop spectrophotometer absorbance readouts of antibody concentration in the supernatant before and after the first wash, with negligible readouts indicating near 100% coating efficiency. Bead Blocking Buffer (Quanterix, 101356) was applied to the beads and incubated on a rotator for 45 min at room temperature. One wash with Bead Wash Buffer then two washes with Bead Diluent Buffer (Quanterix, 100458) followed. The beads were stored at 4°C until use. Quanterix estimate 10% bead loss in the conjugation process, producing antibody‐conjugated bead stocks of 1.26 × 10^9^ beads/ml.

#### 6.5.2 Calibrants

Aβ fibrils were produced following modifications to our previously published protocol [10]. In short, lyophilized recombinant monomeric Aβ peptide (Stratech, A‐1170‐2‐RPE‐1.0 mg) was dissolved on ice in 1x PBS (pH, 7.4) to 200 µM monomer concentration, aliquoted and snap frozen. To generate Aβ fibrils, an aliquot of the dissolved peptide was thawed, vortexed, pulled down for 5 s in a microcentrifuge, diluted to 4 µM in 1x PBS with 0.01% (w/v) sodium azide (Merck, 71290) and incubated for one week at 37°C. The aggregates were sonicated at 20 kHz, 40% power, with a 3‐mm probe‐fitted tip Q125 Sonicator (QSonica, Q125‐110) in an ice‐water bath for 24×5 s pulses with 15 s intermittent rests. Sonicated fibrils were aliquoted and snap‐frozen for storage at −80°C until use. The Aβ peptide monomer concentration was provided by the manufacturer and used as an approximate amyloid‐beta aggregate concentration to calibrate Simoa brain homogenate readouts with 6E10. For tau, lysates from cells stably expressing hyperphosphorylated tau aggregates were used as previously described [5, 10].

#### 6.5.3 Sample Dilutions

Brain homogenate samples were thawed and diluted to 1:20 in Homebrew Detector/Sample Diluent (Quanterix, 101359) for 6E10 assays, or in Tau 2.0 Sample Diluent (Quanterix, 103847) at 1:4000 for AT8 assays and 1:20000 for HT7 assays.

#### 6.5.4 Simoa Loading

To a Simoa 96‐Well Plate (Quanterix, 101457), 100 µl of each pre‐diluted sample or calibrant was added per well with 25 µL capture antibody‐coated beads, equivalent to ∼500 000 beads, and incubated for 30 min on the Simoa shaker at 800 rpm, 30°C, followed by plate washing. All washing steps were performed on the Simoa Microplate Washer with a magnet, using the 3‐step‐assay automated program with Simoa Wash Buffer A (Quanterix, 103078) and B (Quanterix, 103079). 100 µL of 0.3 µg/mL biotinylated detection antibody was added to each well and incubated for 10 mins on the Simoa shaker at 800 rpm, 30°C, followed by another wash cycle. 100 uL of 150 pM streptavidin β‐galactosidase enzyme (SBG, Quanterix, 100439) was added to each well, and the plate was incubated for 10 min at 30°C 800 rpm on the shaker, followed by washing. After two final washes between 1‐min shaking incubations, the Simoa beads were left to dry for 10 min before reading the plate.

#### 6.5.5 Simoa Reading

The plate was inserted into the SR‐X Ultra‐Sensitive Biomarker Detection System (Quanterix). Resorufin β‐D‐galactopyranoside (RGP, Quanterix, 103159) was automatically added to the wells. The RGP had been incubated at 30°C for 2 h beforehand. Automatically, the resuspended beads were moved to Simoa Disc flow cells containing over 200 thousand microwells, each suitably sized for one bead (Quanterix, 100001). The machine used oil to remove excess beads and seal the wells before fluorescence imaging. Microwells containing beads with at least one immunocomplex fluoresced, through SBG cleaving RGP, and the machine calculated this fraction of enzyme‐active fluorescent beads (f_ON_) per well.

#### 6.5.6 Simoa Analysis

As per Simoa conventions, the f_ON_ was converted to average enzymes per bead (AEB). For aggregate specific testing, we ensured AEB was calculated in digital mode, which assumes Poisson statistics for the *number* of immunocomplexes per fluorescent bead. Whereas analog mode measures fluorescence *intensity* of each bead: this is not suitable for aggregate‐specific tests as each aggregate can be bound by multiple enzymes, which affects fluorescence intensity and increases aggregate size bias.

Digital AEB is calculated by:

$$\mathrm{AE}B_{\mathrm{digital}}=-\ln\;\left[1-f_{\mathrm{ON}}\right]$$

where *f*_ON_ > 0.7 (exceeding 70% immunocomplexed beads), the uncertainty from Poisson distributions is considered to rapidly grow. Sample dilutions were selected carefully meaning in this study all *f*_ON_ < 0.9.

To extract approximate aggregate concentrations of the samples, the AEB values were fitted to the calibrant readouts using four‐parameter logistic (4PL) curves.

$$\gamma =D\;+\frac{A-D}{1+{\left(\frac{x}{C}\right)}^{B}}\left(4\mathrm{PL}\;\mathrm{equation}\right)$$

To determine approximate concentrations of monomer in aggregated form in the homogenate, firstly homogenization buffer negative controls were subtracted if these were within the calibration curve range, and then values were multiplied by sample dilution (20,000 for HT7, 4000 for AT8 and 20 for 6E10). For 6E10, nM of calibrant known monomer concentration was converted to ng/ml for the Simoa readouts, by multiplying the normalized nM values by 4.514 kDa, the reported average molecular weight of amyloid‐beta (Abcam, ab120301). The concentrations were divided by BCA‐derived total protein concentration per respective homogenate, to present the proportion of total protein of the homogenate accounted by the detected protein aggregates, enabling approximate comparison between results from different antibodies.

### 6.6 Single Molecule Pull‐Down (SiMPull)

SiMPull experiments are based on prior methods for SiMPull [15] and coverslip passivation [41] optimized from previously published methods for tau aggregates [5].

#### 6.6.1 SiMPull Passivation

Borosilicate glass coverslips of 26 × 76 mm #1.5 (VWR, MENZBC026076AC40) were washed in an Ultrasonic Cleaning Bath (VWR, USC100T) for 10 min cycles in the following solvents: 18.2‐MΩ cm water, acetone, then methanol; before 20 min in 1 M potassium hydroxide. The slides were rinsed with methanol and water, then dried with nitrogen. The coverslips were cleaned with argon plasma for 15 min (Harrick Plasma, PDC‐002), then placed in the ultrasonic bath for salinization with 1.5:2.5:50 of 3‐ Aminopropyltriethoxysilane (APTES) (Thermo Fisher Scientific, 151080000) acetic acid, and methanol, respectively, for two cycles of 1 min sonication and 10 min rest after each cycle. The coverslips were rinsed in methanol, water, then methanol, before drying with nitrogen. A 50‐well gasket with 3 mm × 1 mm wells (Grace Bio‐Labs CultureWell, 103250) was affixed to each clean coverslip. For well passivation, 110 mg/ml methoxy poly(ethylene glycol) succinimidyl valerate (Laysan Bio Inc., MPEG‐SVA‐5000) and 100 mg/ml biotin poly(ethylene glycol) succinimidyl valerate (Laysan Bio Inc., Biotin‐PEG‐SVA‐5000) were mixed in a 1:99 ratio and 9 µL was added to each well with 1 µL of 1 M sodium bicarbonate (NaHCO_3_, pH 8.5) for 24 h incubation at room temperature in a humidity chamber. Then, the coverslips were rinsed with water, nitrogen‐dried before a second round of passivation: 9 µL of 10 mg/ml Methyl‐PEG‐NHS‐Ester (MS(PEG)4) (Thermo Fisher Scientific, 22341) and 1 µL of 1 M NaHCO_3_ (pH 8.5) were added for 24 h humid chamber incubation. The coverslips were rinsed with water, nitrogen‐dried, and vacuum sealed with desiccant for −20°C storage.

#### 6.6.2 SiMPull Loading

Coverslips were equilibrated for 1 h to room temperature, then kept in humidity chambers throughout preparation to reduce well evaporation. After the initial TBST (0.05% Tween 20 in TBS) incubation for 5 min, wells were incubated with 0.2 mg/ml NeutrAvadin in TBST for 10 min before a wash cycle. Each cycle consisted of twice washing with TBST, then once 1% Tween 20 in TBS with 5 min incubation. In all experiments, biotinylated capture antibodies were diluted to 10 nM for 15 min incubation. To reduce non‐specific binding, all tau capture and detection antibodies were diluted in 1 mg/mL BSA in TBS. A capture antibody addition and wash cycle was followed by blocking, involving 1 mg/ml BSA in TBS for 10 min, then one TBST wash. Then brain homogenate samples were added, prediluted to 1 in 10 in TBS and incubated for 1 h. After a wash cycle, Alexa Fluor 647 dye‐labelled detection antibodies were added for 15 min: AT8 antibodies were diluted to 5 nM, and HT7 was diluted to 2 nM. For colocalization experiments, AF647‐labelled AT8 antibody and AF488‐labelled T181 antibody were mixed at equimolar concentrations (2 nM in 1 mg/mL BSA) and incubated for 15 min. Following a wash cycle, and a final addition of TBS to wells, the slides were ready for diffraction‐limited imaging.

DNA PAINT experiments were performed as previously described [16]. Briefly, after sample incubation, a wash cycle was performed, and wells were blocked with 1 mg/mL BSA in TBS for 30 min. Then, DNA‐labelled detection antibody (DBCO TEG‐AAACCACCACCACCACCACCACCACCACCACCACCA, ATDBio) was added (2 nM, 0.1 mg/mL BSA in TBS) for 10 min. After a last wash cycle, the corresponding imaging strand (TGGTGGT – Amino C7 – Atto655, ATDBio) was added (3 µL, 1 nM in PBS). The coverslip was sealed with a clean coverslip.

#### 6.6.3 Microscope Setup

For imaging, a home‐built TIRF microscope was used: an inverted microscope body (Nikon, Ti‐2 Eclipse) with an Apochromat TIRF 60×1.49 numerical aperture oil objective and a perfect focus system. 638 nm laser (HÜBNER Cobolt, 06‐MLD‐638) was used to visualize emission from antibodies with Alexa Fluor 647‐conjugation, and 488 nm laser (HÜBNER Cobolt, 06‐MLD‐488) was used to visualize Alexa Fluor 488‐conjugated T181 antibodies, enabling multi‐color imaging. The fluorescence was passed from the objective through a quad‐band dichroic beam splitter and then emission filters for light emitted from the 488 laser (IDEX Semrock, BLP01‐488R‐25×36 and FF01‐520/44‐25×36) and 638 laser (IDEX Semrock, BLP01‐635R‐25×36) respectively to be captured by the EMCCD camera (Photometrics Evolve 512) in frame‐transfer mode (6.3 electrons/Analog‐Digital Units (ADU) and 250 ADU/photon).

#### 6.6.4 Diffraction‐Limited Acquisition

For colocalization SiMPull imaging, 9 fields of view (FOVs) were taken per SiMPull well, with each FOV imaged for 50 frames of 50 ms exposure, with total internal reflection fluorescence (TIRF) imaging to minimize background noise. Each field of view was 512 × 512 pixels, with each pixel equivalent to 107 nm. Image collection was automated, using Micro‐Manager [42], for sequential capture of images in 488‐ and 638‐ channels, and to avoid FOV location bias.

#### 6.6.5 Diffraction‐Limited Analysis

Images were analyzed with ImageJ plugin, ComDet, controlled by a self‐written macro. This analysis approach was based on previous literature [5]. In brief, the first 5 frames of each FOV were excluded, and the remainder were Z‐stacked per FOV with mean pixel intensities averaged to reduce background noise. For colocalization imaging, the laser channels were merged into composite images per FOV. The thresholds selected for ComDet analysis in this study used a particle size of 4 pixels and an intensity of 8 standard deviations, selected based on signal:noise of AT8 capture and T181 and AT8 detection positive and negative controls, and kept consistent across experiments. The macro extracted the number of particles per FOV and their brightness, in arbitrary units. For colocalization experiments, each detection channel ran through ComDet individually, then colocalized particles were quantified. The maximum Euclidean distance between particles for colocalization was set to a 4 pixel‐radius. By chance colocalization was estimated by repeating this protocol, but with the images from one fluorescence channel rotated 180° before colocalizing with FOV location‐matched second channel images. The colocalized spot proportion as a proxy for the proportion of multisite phosphorylated aggregates was calculated within ComDet, by dividing colocalized particle quantities by total particle quantities per fluorescence channel.

#### 6.6.6 Super‐Resolution Image Acquisition and Analysis

For DNA PAINT imaging, imaging was performed using a 638 nm laser at 190 mW. For each well, typically 4 FOVs were collected in a 2‐by‐2 grid using an automated script (*Micro‐Manager*) [43] to avoid any bias in the selection of FOVs. Images were acquired for 8000 frames of 100 ms exposure.

Super‐resolution images were reconstructed using the *Picasso* package [44]. After discarding the first 100 frames, localisations were identified, fitted, and then drift corrected using redundant cross‐correlation. Localisations were then filtered for precision <30 nm and clustered using *DBSCAN* through the *scitkit‐learn* package [45] (radius of 0.3 (∼35 nm) and minimum density of 5). Further characteristics of each aggregate were then measured using scikit‐image [46] for basic region properties such as perimeter, area, and eccentricity, and *SKAN* for skeletonized length (whereby the length of each aggregate is reported as the summed branch distance). The super‐resolved images were rendered using the inbuilt *Picasso* functionality.

#### 6.6.7 Single‐Molecule Statistical Analysis

For statistical tests, MatLab codes were written to run Wilcoxon rank‐sum tests upon mean average values per donor within Braak stage groups 0–2, 3–4 and 5–6, and Kolmogorov‐Smirnov tests with Bonferroni correction were applied to cumulative frequency values within the matched Braak stage groupings. Non‐parametric tests were selected to not assume normal distribution or balanced sample sizes. To explain technical repeat differences, error bars were calculated using ±1 standard error, for example between FOVs or between Simoa experiment repeats. To propagate this error, for example between multiple microscopy experiments, absolute error was calculated, whereby d = standard error:

$$\begin{array}{ccc} d\left(A+B+C\text{…}\right) & = & \left(\mathrm{sqrt}\left({\left(\mathrm{dA}\right)}^{2}+{\left(\mathrm{dB}\right)}^{2}+{\left(\mathrm{dB}\right)}^{3}\text{…}\right)\right)\\ & & /\mathrm{count}\left(A:C\text{…}\right) \end{array}$$

When incorporating multiple experiment types into means, such as BCA normalization, or making analysis ratios, the error was further propagated, whereby d = absolute error:

$$d\left(A/B\right)=\left(A/B\right)\left(\mathrm{sqrt}\;({\left(d\left(A\right)/A\right)}^{2}+{\left(d\left(B\right)/B\right)}^{2})\right)$$

### 6.7 Bulk RNA Extraction, Sequencing, Pre‐Processing and Differential Gene Expression Analysis

Samples were randomized for RNA extraction to reduce batch effects between conditions. Trizol was added to homogenize tissue, followed by Chloroform. Rigorous vortexing of the samples resulted in homogenization. A Qiagen RNeasy micro kit (cat no 74004) was used for extraction and purification as per the manufacturer's instructions. Briefly, samples in MinElute Spin Columns were treated sequentially with buffers RW1 and RPE. Samples underwent DNase I digestion, and the resulting RNA was eluted in RNase‐free water. Samples were quantified using the nanodrop, and RIN values were obtained using an Agilent Bioanalyzer (RNA nano chip and reagents, 5067‐1512).

Samples were normalized for concentration and underwent ribodepletion using the New England Biolabs rRNA depletion kit (E7400X). Genomic Libraries were constructed using the New England Biolabs Library construction kit (E7760L), using the NEBNext Multiplex oligos for Illumina (E6442S). Libraries were quantified using a Qubit fluorometer and pooled for sequencing using a HiSeq4000 at 50 million reads per sample. Samples were split across multiple lanes to avoid batch effects. The bulk RNA‐seq data were preprocessed using the nextflow pipeline nf‐core‐rnaseq v3.10.1 using default parameters, with alignment and quantification performed by the included STAR and RSEM tools. The postmortem tissue samples were rejected during quality control if less than 90% of reads could be aligned, with a minimum requirement of 30 million aligned reads, resulting a total of 25 samples.

Differential gene expression analysis was performed using **DESeq2** (v 1.44.0 [47]), comparing samples with neuropathologically defined AD (Braak stage ≥ 3) and Control (Braak stage < 3), incorporating a model adjusting for sex, brain region, ApoE allele status and RNA integrity number (RIN). To reduce the influence of spurious fold changes arising from genes with low mean expression, log2 fold change (log2FC) shrinkage was subsequently applied using the apeglm algorithm as implemented in DESeq2. Genes with an absolute shrunken log2FC ≥ 0.5 and an adjusted *p*‐value ≤ 0.05 (FDR) were considered significantly differentially expressed, and only significant protein coding‐genes used for pathway enrichment analysis. For visualization, a volcano plot was generated using raw (pre‐shrinkage) log2FC values, with the baseMean ≥ 10. Only top 10 protein‐coding genes significant by padj are labelled on the volcano plot.

### 6.8 Weighted Gene Co‐Expression Network Analysis

A weighted gene co‐expression (WGCNA) [22] network bulk RNA sequencing data was generated from a transformed and covariate‐corrected gene count matrix. The raw gene count matrix was transformed using variance‐stabilizing transformation (vst) using the “vst” function from R package DESeq2 prior to batch correction. Batch correction was performed using the “removeBatchEffect” from R package limma to regress out the effect of RIN, post‐mortem delay, age and sex. This transformed and batch‐corrected gene count matrix was used as the input of network analysis using the WGCNA algorithm (version 1.72.5). Network construction used all 25 samples (12 MTG and 13 SOM). Sampling two regions per donor was a deliberate design choice, as the two regions differ substantially in pathological burden and together provide the range of variation required for co‐expression network inference; constructing networks within each region separately would leave 12 and 13 samples, below the sample size at which WGCNA inference is considered reliable. The network construction uses expression data only, with no trait variables, therefore, the pairing of samples within donors does not bias module assignment. A signed hybrid network was built with the following settings: soft threshold power = 10, minimum module size of 30, deepSplit = 1. Specifically, first, pairwise Pearson correlations between each gene pair were calculated, and this correlation matrix was transformed into a signed adjacency matrix. Next, the connection strength of components within this matrix was used to calculate a TOM (topologically overlapping matrix), which represents measurements of gene expression pattern similarity across samples constructed on the pairwise correlations for all genes within the network. Then, hierarchical gene correlation clustering was done using 1‐TOM, and finally, dynamic tree cutting was used to split the modules. This resulted 27 modules consisting of 30 or more genes. Genes which could not be assigned to any modules were placed into module M0.

### 6.9 Module–Trait Correlations of the WGCNA Modules

Pearson correlations were calculated between module eigengenes and traits of interest to prioritize biologically relevant modules. The two regional samples from each donor are not statistically independent, these correlations were computed within each region separately, so that every donor contributes a single observation. Module eigengenes were calculated once on the full sample set and subsequently subset by region, so that module definitions remained fixed across analyses. Correlations computed across all samples are shown alongside the region‐stratified analyses in Figure S6 for comparison but were not used to support the associations reported in the text.

### 6.10 Stability of Module–Trait Correlations

The robustness of each module–trait correlation was assessed within each region by leave‐one‐donor‐out analysis, in which the correlation was recomputed omitting one donor at a time, and by donor‐level bootstrap resampling (5000 replicates) to obtain 95% confidence intervals. Associations were considered directionally stable if the sign of the correlation was unchanged across all leave‐one‐donor‐out fits.

### 6.11 Module Preservation

To assess whether module structure was reproducible outside the discovery cohort, module preservation statistics were computed using the module Preservation () function in WGCNA, with the discovery cohort as the reference network and an independent, previously unpublished bulkRNAseq MTG dataset generated in our laboratory as the test network (Table S3, *n* = 38 donors with no sample overlap with the discovery cohort). Analyses used 100 permutations, with randomly sampled (“gold”) and unassigned (“grey”) gene sets as internal references. Preservation was assessed using Zsummary and median rank statistics; Zsummary scales with module size, therefore, median rank was used for comparison between modules of differing size.

### 6.12 Single Nuclei Transcriptomics

Single nucleus isolation was performed following established protocols [26, 48]. Two sets of snRNAseq data were generated from the same set of brains. Firstly, microglia and astrocytes were enriched by depleting neuronal and oligodendrocyte nuclei using Fluorescence‐activated Sorting (FACS) (Glia Enriched). In the second set, all cells from the brain were sampled (total population). Both sets underwent the same library preparation and sequencing protocols. Approximately 7000 isolated nuclei per sample were processed and sequenced using the 10× Genomics Chromium Single Cell 3' platform. Preprocessing, quality control, integration, clustering, and cell type assignment of snRNA sequencing data were conducted as described in^3^. The present analysis focuses on microglia astrocyte from the enriched set, and the neuronal subset from the total population. snRNAseq data were visualized using the dimensionality reduction method UMAP (Uniform Manifold Approximation and Projection) [49]. In addition to our in‐house generated snRNAseq data, we utilized a large‐scale published snRNAseq Dataset comprising 449 samples from the Dorsolateral Prefrontal Cortex (DLPFC) spanning all Braak stages, derived from the ROSMAP cohort [28]. The original cell‐type annotations provided by the authors were retained to ensure consistency and generalizability. Raw or processed count matrices were obtained from https://www.synapse.org/synapse:syn31512863. No additional clustering or re‐annotation was performed. Microglial subcluster proportions were computed from the original cell‐type annotations and used for the correlational analysis against pathological burden shown in Figures S9 and S10. For mediation analysis, quantitative measurements of amyloid‐beta plaque burden and tau tangle load provided alongside the published dataset were used directly as trait variables, consistent with the approach applied to our in‐house cohort. Differential gene expression analysis in single nuclei transcriptomics

We used model‐based analysis of single‐cell transcriptomics (MAST) to identify genes differentially associated with histopathological features, using each feature as a dependent variable in a zero‐inflated regression analysis using a mixed‐model [50]. Models were fit separately for each cell type. The model specification was *zlm(∼Pathology_features+ (1|sample) + cngeneson + pc_mito + sex + age + post mortem interval + Brain region + ApoE*, method = “glmer,” ebayes = F). The fixed‐effect term cngeneson is the cellular detection rate as previously described, and pc_mito accounts for the relative proportion of counts mapping to mitochondrial genes. Each model was fit with and without the dependent variable and compared using a likelihood ratio test. Genes expressed in at least 10% of cells were evaluated for gene expression. The threshold for significant differential gene expression was a log2 fold‐change of at least 0.25 and a Benjamini‐Hochberg adjusted *p*‐value (padj) < 0.05.

### 6.13 Pathway Enrichment Analysis

Pathway enrichment was performed using the enrichR (v 3.3) package. Statistically significant differentially expressed genes were submitted for over‐representation analysis against the “GO_Biological_Process” and “Wikipathways” databases. The false‐discovery rate (FDR) was calculated using the Benjamini‐Hochberg method, and filtering was applied at a significance threshold of ≤0.05. Pathways were then selected based on the removal of pathways with fewer than 2 overlapping genes. During visualization, to reduce redundancy arising from overlapping gene sets within databases, pathways were clustered based on gene overlap, and the top 2 lowest‐FDR pathways per cluster were retained. For neuronal pathway enrichment analysis in snRNAseq data, genes expressed in 10% of neuronal nuclei were set as the background gene set.

### 6.14 Mediation Analysis

Mediation analysis was performed to identify genes that regulate tau aggregate formation. Following the chain of AD pathogenesis that starts with amyloid‐beta aggregates, leading to tau accumulation [51], we tested whether gene expression can mediate amyloid‐beta‐driven tau aggregate. Models were fitted utilizing snRNAseq microglial data transformed to sample‐level pseudobulked expression, with amyloid‐beta and tangle load measured once for each donor brain. The primary analysis was restricted to MTG. Thus, each of the 13 donors contributes only a single sample; donor‐level bias does not arise. MTG was selected as the primary region because it showed a stronger amyloid–tangle association than did the SOM; tangle load was at or near the detection floor in the majority of SOM samples.

Covariates were specified a priori. A variable was included only if it was a risk factor for both amyloid‐beta and tangle load and not a consequence of either. Age at death was retained as Age is positively associated with both amyloid‐beta and tangle load within each region (Figure S10A). Braak stage and diagnosis were excluded as consequences of the pathology under study, and APOE genotype because it lies upstream of amyloid‐beta deposition. Four nested specifications (unadjusted; +age; +age and sex; +age, sex and APOE) are reported in Table S15 where the age‐adjusted model is presented as primary. The mediator model was “gene expression ∼ G_AB + covariates” and the outcome model “AggregateConc_AT8 ∼ gene expression + G_AB + covariates”. Exposure and outcome were standardized to unit variance, so that the average causal mediation effect is expressed in outcome standard deviations per exposure standard deviation. Indirect effects were estimated as the product of coefficients, algebraically identical to the *mediate()* function of the R package mediation for linear models without an exposure–mediator interaction; a vectorized implementation was used to permit genome‐wide resampling and was verified against the package (maximum absolute difference in ACME < 1 × 10^−^^14^). Confidence intervals were obtained from 5,000 percentile bootstrap replicates in the discovery cohort and 10 000 in the replication cohort. No gene survived FDR correction under any specification in the internal dataset, and results are interpreted on effect size, direction and stability across specifications rather than significance thresholds.

For completeness, pooled two‐region estimates (*n* = 25, both MTG and SOM from 13 donors) also were derived using bootstrap resampling of both donors and samples separately, under the same covariate specification (BrainRegion + Age). The confidence intervals widened for 78% of genes in the donor‐level bootstrap (median width ratio 1.11, Figure S10C), confirming that sample‐level resampling understates uncertainty in the presence of intra‐donor correlation. These pooled results are presented as a sensitivity analysis only (Table S15).

To test the microglial component of this signal with greater power, the same procedure was applied to snRNAseq data from a previously published large‐scale dataset [28], within the microglial clusters Mic.12 (*n* = 415 donors) and Mic.13 (*n* = 337). Analyses were adjusted for “age at death” and were restricted a priori to genes belonging to the M12 co‐expression module (76 of which were represented in that dataset), allowing false discovery rate correction within a hypothesis‐driven gene set rather than across the transcriptome. M12 was defined by co‐expression clustering in our bulk RNAseq data, without reference to pathology measures or to any mediation result, so this restriction is independent of the discovery analyses. FDR correction was applied within the restricted gene set, and significance is reported at FDR < 0.1.

### 6.15 Fitting a Model for Aggregate Length Distribution

The quantitative model capturing the key features of disease was developed in [17] and is expected to be valid for large aggregates. We therefore only fit the model to aggregates within a size region, aggregates sized between *nmin* and *nmax* monomers. We determine these values based on the observed aggregate length distribution, see Table 2 for values. To convert from measured length to monomers, we use the conversion of 4 monomers per nanometer, assuming a double‐stranded aggregate with 0.5 nm beta‐sheet separation [52].

**TABLE 2:** Fitting regime for the quantitative model across samples.

Region	Antibody	Stage	nmin	nmax
MTG	AT8	Control	320	2400
MTG	AT8	1‐2	320	2400
MTG	AT8	3‐4	320	3000
MTG	AT8	5‐6	320	5600
SOM	AT8	Control	320	2000
SOM	AT8	1‐2	320	1800
SOM	AT8	3‐4	320	2400
SOM	AT8	5‐6	320	4400
MTG	HT7	Control	400	1000
MTG	HT7	1‐2	400	1400
MTG	HT7	3‐4	400	2400
MTG	HT7	5‐6	400	4000
SOM	HT7	Control	400	1400
SOM	HT7	1‐2	400	1400
SOM	HT7	3‐4	400	1800
SOM	HT7	5‐6	400	3200

We fit using a two‐step Bayesian Inference process to determine the mechanistic rates governing the observed aggregate length distribution. Firstly, we fit the distribution of aggregates in the control samples to determine the geometric decay factor in healthy cells, α_*H*_. The likelihood of observing an aggregate of size *x* monomers is

$$p\left(x,\alpha_{H}\right)=\frac{\alpha_{H}^{x}\left(1-\alpha_{H}\right)}{\alpha_{H}^{nmin}-\alpha_{H}^{nmax+1}}$$

We determine a posterior distribution of α_*H*_ for each control sample (both antibodies in both regions) and fit all repeats together.

Secondly, we use the maximum likelihood value α_*H*_ and consider the geometric decay factor in pathological cells, α_*D*_, and the proportion of fitted aggregates from pathological cells, β, across individuals with disease. The likelihood to observe an aggregate of size *x* from either cell state is

$$p\left(x\right)=\beta p\left(x,\alpha_{D}\right)+\left(1-\beta \right)p\left(x,\alpha_{H}\right)$$

As discussed in the main text, the relative removal rate is lower by a factor *r_c_* in pathological cells, and we can connect this to the geometric decay factors via

$$r_{c}=\frac{\alpha_{D}^{-1}-1}{\alpha_{H}^{-1}-1}$$

To determine the parameters describing the aggregate distributions from disease, we perform a Bayesian inference of *r_c_* and β with a uniform prior between 0 and 1 for β and between 0 and 0.9, for *r_c_*. The upper limit of 0.9 ensures that the two cell states are sufficiently different, i.e., that a second population is only assigned when the relative removal rate has been decreased by at least 10%. If the removal rate were increased, i.e., *r_c_* > 1, then the length distributions in disease would have to be shorter‐tailed than in control, which is clearly inconsistent with the observed distributions.

We group the samples by Braak stage (1‐2, 3–4, 5–6) and perform this procedure on combined aggregate length distributions from all repeats. We also performed the same analysis on the data from each sample individually, see Figures S20–S24, which show the same trend but only provide poor constraints on the relative removal in samples with a low fraction of diseased cells, *β*, due to the under‐sampling of the largest aggregates when only a single sample is used. For either both analyses, as the resulting values for *r_c_* and β were obtained by fitting a model, they are naturally conditional on the assumptions made to obtain this model.

## Author Contributions

**Elizabeth A. English**: conceptualization, methodology, writing – original draft, writing – review and editing, project administration, data curation, formal analysis, investigation, visualization. **Johanna S. Jackson**: methodology, writing – review and editing. **Georg Meisl**: writing – review and editing, methodology, software, formal analysis, visualization. **John S. H. Danial**: methodology. **Paul M. Matthews**: conceptualization, funding acquisition, writing – review and editing, project administration, supervision. **Nurun Fancy**: conceptualization, methodology, writing – original draft, writing – review and editing, project administration, data curation, formal analysis, investigation, visualization. **David Klenerman**: conceptualization, writing – original draft, writing – review and editing, project administration, funding acquisition, supervision. **Dorothea Böken**: methodology, software, investigation, writing – review and editing, data curation, formal analysis, visualization. **Matthew R. Cheetham**: methodology. **Matthew W. Cotton**: software, methodology, writing – review and editing, formal analysis, visualization.

## Conflicts of Interest

The authors declare no conflicts of interest.

## Data Presentation

Cumulative frequency and super‐resolution eccentricity by length scatter plots were made using MatLab (Version R2022b). Figures 5, 6, 7 and Figures S6–S15 were generated using R (Version 4.2.2. All other graphs were produced using Microsoft Excel (Version 2205). Methodological diagrams were made using Biorender.

## Data and Code Availability

All original code for the super‐resolution analysis has been deposited at Zenodo at https://doi.org/10.5281/zenodo.15635269 and is publicly available as of the date of publication. All data are available in the manuscript orthe supplementary material. while the transcriptomics data can be found at: https://www.synapse.org/Synapse:syn52658340. Raw data is available on written request to DK.

## Supporting information




**Supporting File 1**: advs77453‐sup‐0001‐SuppMat.docx.

**Supporting File 2**: advs77453‐sup‐0002‐SuppMat.xlsx.

## Data Availability

The data set that supports these findings have been made publically available.
