# Supplementary material for: Tau Aggregate Imaging and Transcriptomics of Alzheimer's Disease Brain at Different Stages of Disease
Source: Adv Sci (Weinh). 2026 Aug 31:e77453. Online ahead of print. doi: 10.1002/advs.77453 (PMC13528684; doi:10.1002/advs.77453)
Supplement: Supplementary file 1 [file ADVS-9999-e77453-s001.docx]

**SUPPLEMENTARY**

**Supplementary Figure S1:** White matter immunohistochemistry measures of amyloid-beta (4G8) and phosphorylated tau (AT8 and PHF1) area in post-mortem brain tissue.

**Supplementary Figure S2: Additional diffraction-limited SiMPull colocalisation brightness analysis.**

A. Mean intensity of colocalised compared to non-colocalised spots in MTG and SOM homogenate across Braak stages.

B. Intensity ratio of colocalised versus non-colocalised spot means showed colocalised spots had greater intensities in most samples. The ratio significantly increased for AT8-T181 with progressive Braak stages.

C. Proportion of bright spots (>500 arbitrary units) increased with Braak stage.

D. Maximum averaged spot intensity per sample increased with Braak stage, significantly for AT8-T181 colocalised and non-colocalised spots.

E. Minimum averaged intensity was significantly higher in colocalised than non-colocalised spots.

A-E: Each spot represents mean average results from one donor and error bars denote ± 1 absolute error from standard errors, incorporating intra-plate error between 9 FOVs in each experiment, and inter-plate error between 3 experimental repeats. Wilcoxon rank-sum test between Braak stage groups 0-2, 3-4 and 5-6, * p<0.05, *** p<0.005, **** p<0.001.; n.s., non-significant.

**Supplementary Figure S3: Additional super-resolution SiMPull aggregate size and shape analysis.**

A. Mean cluster length, in nanometres.

B. Eccentricity of clusters, with 0 being circular and 1 being linear.

C. Percentage of short, round aggregates (clusters < 100 nanometres, eccentricity <0.7).

A-C: Each spot represents mean average results from one donor and shading denotes S.D. across patients. Wilcoxon rank-sum test with Holm correction between Braak stage groups 0-2, 3-4 and 5-6. * p < 0.05, ** p < 0.01. Only significant comparisons are shown.

**Supplementary Figure S4: Cumulative frequency of tau (HT7+) and AT8+ p-tau aggregate length and eccentricity.**

A: Cumulative frequency plots of aggregate length, in nanometres.

B: Cumulative frequency plots of aggregate shape, based on eccentricity, with 0 representing completely circular clusters and 1 representing completely linear clusters.

A,B: MTG: Middle temporal gyrus, SOM: somatosensory cortex. Each line incorporates all clusters per grouped Braak stages, shading indicates S.D. between patients.

**Supplementary Figure S5: Differential gene expression analysis in bulkRNA sequencing data.**

A: Volcano plot showing significantly up- (logFC ≥ 0.5, padj ≤ 0.05) and down-regulated (logFC ≤ −0.5, padj ≤ 0.05) genes between AD (Braak ≥ 3) and Control (Braak < 3) samples. The logFC represents raw logFC prior to “apeglm” shrinkage. Genes with a baseMean lower than 10 were removed for the visualisation. Top 10 significant protein-coding genes by padj are labelled. logFC, log2 fold-change; padj, adjusted p-value.

B: B. Bar plot showing pathway enrichment analysis of differentially up-regulated protein-coding genes. Genes with a shrunk logFC **≥** 0.5, padj ≤ 0.05 were considered as significant and used for enrichment analysis. Top pathways were selected applying the following criteria sequentially (1) a minimum overlap of 2 genes between the query gene list and the geneset; (2) an FDR ≤ 0.05; (3) selection of the top 2 lowest-FDR pathways per cluster (where pathways were clustered based on gene overlap to reduce redundancy); and (4) visualisation of the top 12 pathways overall by FDR across both databases. Fill colors showing the -log10 transformed FDR of the significance p-value. FDR, false-discovery rate.

**Supplementary Figure S6: Module-trait correlation computed across all samples and within each brain regions.** Pearson correlations between module eigengenes (M1–M27) and pathological, neuropathological and demographic traits. Student asymptotic p-value was calculated for Pearson’s correlations (*p≤0.05, **p≤0.01, ***p≤0.001).

**A:** All samples pooled (n = 25; 12 MTG and 13 SOM).

**B:** MTG only (n = 12).

**C:** SOM only (n = 13).

**Supplementary Figure S7: Stability of module–trait correlations and preservation of module structure.**

**A:** Robustness of module–trait correlations within each brain region (MTG, n = 12; SOM, n = 13). Each point represents one module–trait pair. The x-axis gives the absolute correlation coefficient from the full sample, and the y-axis the width of its 95% confidence interval obtained by bootstrap resampling of donors (5,000 replicates). Point colour indicates whether the confidence interval excluded zero (red) or included it (grey); point shape indicates whether the direction of the correlation changed in any leave-one-donor-out fit (cross) or was consistent across all fits (circle). Confidence interval width is approximately constant across the range of effect sizes, indicating that associations with larger absolute correlations are more robust because they lie further from zero rather than because they are more precisely estimated. Labelled points are those with |r| > 0.5 that were both directionally stable and had confidence intervals excluding zero.

**B:** Preservation of discovery modules in an independent bulk RNA-seq MTG dataset (n = 38 donors, no sample overlap with the discovery cohort), computed with 100 permutations. Each point represents one module; randomly sampled ("gold") and unassigned ("grey") gene sets are included as internal references (triangles). Zsummary is plotted against module size, with dashed and dotted lines marking conventional thresholds for strong (Z > 10) and weak (Z > 2) preservation. Because Zsummary scales with module size, the high values for the two reference sets reflect their size (n = 1,000 genes each) rather than genuine preservation; on the size-independent median rank statistic both reference sets rank last.

**Supplementary Figure S8: Distribution of module eigengene and aggregate concentration of different AD associated pathology against Braak stages.** AggregateConc, Simoa derived concentration measured; G_AB, 4G8 positive immunostained area in grey matter.

**Supplementary Figure S9: Gene module membership strength and gene correlation with traits identifies top genes associated with traits.**

A: Scatter plot showing the gene module membership in X-axis and the gene significance of M12 genes for total tau aggregate concentration measured by HT7 in Y-axis.

B: Scatter plot showing the gene module membership in X-axis and the gene significance of M12 genes for p-tau aggregate concentration measured by AT8 in Y-axis.
The module membership is defined as a "fuzzy" measure of module membership by correlating its gene expression profile with the module eigengene of a given module[1]. Gene significance is defined as the correlation of gene expression profiles with an external trait*.*

**Supplementary Figure S10: Amyloid–tangle association, mediation analysis in MTG, and effect of resampling unit on interval estimates.**

**A:** Association between amyloid-β burden (G_AB) and tangle load (AggregateConc_AT8) within each brain region (MTG, n = 13; SOM, n = 12). Lines show linear fits with 95% confidence bands. Spearman correlations are given in each panel with p-values Bonferroni-corrected across the two regions. The association was strong in MTG (rho = 0.89, corrected p = 1.2 × 10⁻⁴) and weaker and non-significant in SOM (rho = 0.52, corrected p = 0.17).

**B:** Mediation analysis within MTG (n = 13 donors, one sample per donor), adjusted for age at death. Each point represents one of 5,366 genes tested, plotted as the average causal mediation effect (ACME, in outcome standard deviations per exposure standard deviation) against −log₁₀ of the bootstrap p-value. No gene survived false discovery rate correction.

**C:** Effect of the bootstrap resampling unit on confidence interval width in the pooled two-region analysis (25 samples from 13 donors, adjusted for brain region and age). Each point represents one gene. Axes give the width of the 95% confidence interval for the ACME under resampling of individual samples (x) and of whole donors (y); the dashed line indicates equality. The confidence intervals widened for 78% of genes under donor-level resampling (median width ratio 1.11).

**Supplementary Figure S11: Pathway enrichment of modules associated with neuronal transcriptome.** Heatmap showing top enriched pathways from modules M14, M15, M17, M19, M20, M21 and M22. Pathways are grouped based on their syntactic similarities.

**Supplementary Figure S12:** Correlations of microglia subclusters as annotated in [2]with tissue amyloid immunostained area.

**Supplementary Figure S13:** Correlations of microglia subclusters as annotated in[2] with tissue tangles immunostained area.

**Supplementary Figure S14**:

A: Average proportion of microglia subclusters as annotated in [2] against Braak stages.

B: Dotplot showing percentage of cells expressing the average scaled expression of marker geneset for each microglia sub-cluster.

**Supplementary Figure S15:** A: Volcano plot visualising the microglial genes having a significant but partial mediation effect on the tissue tangles load using published snRNAseq data [2]. X-axis showing ACME [effect of the mediation (gene expression)] and Y-axis showing the -log10(pvalue). Causal modelling framework where tissue amyloid-beta load is placed as the initiator of AD cascade which triggers gene expression change and the gene expression mediates the increase in tangles burden.

**Supplementary Figure S16:** Upset plot showing the overlap of differentially expressed genes across different neuronal sub-populations in *MidVsEarly, LatevsEarly and LatevsMid* comparisons.

**Supplementary Figure S17: Pathway enrichment of up-regulated genes in mid (Braak 3-4) and late (Braak 5-6) AD samples compared to that of Control (Braak 0-2) samples in neuronal sub-population.** Heatmap showing top enriched pathways from up-regulated genes (defined as log fold-chage ≥ 0.25, padj ≤ 0.05) in each neuronal sub-population. groupMid and groupLate showing results from mid (Braak 3-4) and late (Braak 5-6) AD samples respectively compared to Control (0-2) samples. Pathways are grouped based on their syntactic similarities. Colour bar showing false-discovery rate corrected (FDR) p-value.

**Supplementary Figure S18: Pathway enrichment of down-regulated genes in mid (Braak 3-4) and late (Braak 5-6) AD samples compared to that of Control (Braak 0-2) samples in neuronal sub-population.** Heatmap showing top enriched pathways from down-regulated genes (defined as log fold-chage ≤ −0.25, padj ≤ 0.05) in each neuronal sub-population. groupMid and groupLate showing results from mid (Braak 3-4) and late (Braak 5-6) AD samples respectively compared to Control (0-2) samples. Pathways are grouped based on their syntactic similarities. Colour bar showing false-discovery rate corrected (FDR) p-value.

**Supplementary Figure S19:** Scatter plot showing Pearson’s correlations between MeanBeta_HT7 calculated in Fig. 5 and relative proportion of neuronal sub-populations. R shows absolute correlation coefficient and p denotes p-values from student t-test.

**Supplementary Figure S20:** Bar chart showing maximum likelihood fits for the two-population model combined within Braak stage groups. The individual points shown in black indicate the fits at the individual sample level and this is consistent with the behaviour of the combined data. In samples with a low fraction of diseased cells, the value of rc is more poorly constrained due to under sampling. Note that these fits are the maximum likelihood values and correspond to the fits in figure S21-S24.

**Supplementary Figure S21:** Histograms of measured length distributions for AT8 MTG, with the red line showing the maximum likelihood fit based on the two-population model. The nmin and nmax values are constant within each Braak stage and can be seen from the plot.

**Supplementary Figure S22:** Histograms of measured length distributions for AT8 SOM, with the red line showing the maximum likelihood fit based on the two-population model. The nmin and nmax values are constant within each Braak stage and can be seen from the plot.

**Supplementary Figure S23:** Histograms of measured length distributions for HT7 MTG, with the red line showing the maximum likelihood fit based on the two-population model. The nmin and nmax values are constant within each Braak stage and can be seen from the plot.

**Supplementary Figure S24:** Histograms of measured length distributions for HT7 SOM, with the red line showing the maximum likelihood fit based on the two-population model. The nmin and nmax values are constant within each Braak stage and can be seen from the plot.

[1]	P. Langfelder, S. Horvath, *BMC Bioinformatics* **2008**, *9* (1), 559, <https://doi.org/10.1186/1471-2105-9-559>.

[2]	G. S. Green, M. Fujita, H.-S. Yang, M. Taga, A. Cain, C. McCabe, N. Comandante-Lou, C. C. White, A. K. Schmidtner, L. Zeng, A. Sigalov, Y. Wang, A. Regev, H.-U. Klein, V. Menon, D. A. Bennett, N. Habib, P. L. De Jager, *Nature* **2024**, *633* (8030), 634, <https://doi.org/10.1038/s41586-024-07871-6>.
